# A study on Ti-doped Fe_3_O_4_ anode for Li ion battery using machine learning, electrochemical and distribution function of relaxation times (DFRTs) analyses

**DOI:** 10.1038/s41598-022-08584-4

**Published:** 2022-03-22

**Authors:** Po-Wei Chi, Tanmoy Paul, Yu-Hsuan Su, Kai-Han Su, Cherng-Yuh Su, Phillip M. Wu, Sea-Fue Wang, Maw-Kuen Wu

**Affiliations:** 1 Institute of Physics, Academia Sinica, 128, Section 2, Academia Road, Taipei, 11529 Taiwan; 2 Department of Engineering and System Science, National Tsing Hua University, 101, Section 2, Kuang-Fu Road, Hsinchu, 300044 Taiwan; 3 Institute of Manufacturing Technology and Department of Mechanical Engineering, National Taipei University of Technology (TAIPEI TECH), 1, Section 3, Zhongxiao E. Road, Taipei, 106 Taiwan; 4 Department of Materials and Mineral Resources Engineering, National Taipei University of Technology (TAIPEI TECH), 1, Section 3, Zhongxiao E. Road, Taipei, 106 Taiwan

**Keywords:** Energy storage, Batteries, Batteries, Electronic properties and materials

## Abstract

Among many transition-metal oxides, Fe_3_O_4_ anode based lithium ion batteries (LIBs) have been well-investigated because of their high energy and high capacity. Iron is known for elemental abundance and is relatively environmentally friendly as well contains with low toxicity. However, LIBs based on Fe_3_O_4_ suffer from particle aggregation during charge–discharge processes that affects the cycling performance. This study conjectures that iron agglomeration and material performance could be affected by dopant choice, and improvements are sought with Fe_3_O_4_ nanoparticles doped with 0.2% Ti. The electrochemical measurements show a stable specific capacity of 450 mAh g^−1^ at 0.1 C rate for at least 100 cycles in Ti doped Fe_3_O_4_. The stability in discharge capacity for Ti doped Fe_3_O_4_ is achieved, arising from good electronic conductivity and stability in microstructure and crystal structure, which has been further confirmed by density functional theory (DFT) calculation. Detailed distribution function of relaxation times (DFRTs) analyses based on the impedance spectra reveal two different types of Li ion transport phenomena, which are closely related with the electron density difference near the two Fe-sites. Detailed analyses on EIS measurements using DFRTs for Ti doped Fe_3_O_4_ indicate that improvement in interfacial charge transfer processes between electrode and Li metal along with an intermediate lithiated phase helps to enhance the electrochemical performance.

## Introduction

Rechargeable lithium-ion batteries (LIBs) have attracted continuous attention due to their outstanding properties including high energy efficiency, lack of memory effect, long cycle life, high energy and high power density^1–3^. It has been considered as the primary power source for portable electronic devices, hybrid electric vehicles (HEV) and plug-in hybrid electric vehicles (PHEV)^4,5^. Graphite has been widely used as the anode material in LIBs because of its layered structure, which allows lithium to be inserted/extracted during the charging and discharging processes, that gives a theoretical specific capacity of 372 mAh g^−1^. However, a relatively low reversible capacity and poor cycle stability at a higher rate limit its use in HEV and PHEV^5^, where applications need to meet high energy and high power density.

To find a possible replacement to the graphite, transition metal oxides such as NiO^6,7^, Fe_3_O_4_^8,9^, Fe_2_O_3_^10–12^, SnO_2_^13^, Co_3_O_4_^14^, and CuO^15^ have been investigated owing to their capability to intake excess Li^+^-ion^16^ during the charging and discharging processes that lead to high theoretical capacity (∼700—1000 mAh g^−1^). Among these metal oxides, Fe_3_O_4_ anode based LIBs have been well-investigated because of their high energy, high capacity, environmental compatibility and element abundance. Unfortunately, LIBs based on Fe_3_O_4_ suffer from particle aggregation during charge–discharge processes that affects the cycling performance. It is well documented that the structure of the electrode is difficult to maintain after several charge–discharge cycles^17^. Therefore, extensive studies have been carried out using Fe_3_O_4_ nanoparticles modified with carbon in its various forms including sheet^18^, sphere^19^, nanotube^20^ and carbon fiber^21^, to stabilize the cycle-life and rate capacity. Graphene based Fe_3_O_4_ nanocomposites as anode material in LIBs show enhancement in electrochemical properties with a capacity loss of 5% after 100th cycles at 1 C rate^22^. On the other hand, the report on the effect of the third-element doping to Fe_3_O_4_ showing enhanced electrochemical performance is relatively scarce. A notable example is the Fe_3_O_4_-based Fe_3_O_4_/Cu nanocomposite, which shows high cycle reversibility performance up to 500 cycles at 300 mAh g^−1^^23^.

To understand the origin of the large capacity fading after the first few cycles and how the SEI affects the battery performance, we have carried out a detailed study^24,25^ on LIBs using lithium ferrite (α-LiFeO_2_) as the anode. Structural analyses based on Rietveld refinement of lithium ferrite suggest the existence of two-dimensional lithium-ion migration pathways inside the lattice^24^. The study showed that both Li^+^ and Fe^3+^ occupy the same site in octahedra with almost similar occupancies as determined from structural analysis. Both the magnetic and *ex-situ* XRD studies suggest a structural transformation after the charge–discharge reaction. DFRT (Distribution Function of Relaxation Time) studies based on impedance measurements also justify the structural phase transformation from the 4th cycle onwards.

These studies have correlated the structural, magnetic, and electrochemical measurements to explain the lithium-ion migration and performance degradation of LiFeO_2_. A reversible cycle with a capacity close to ~ 530 mAh g^−1^ at the charge rate of 0.1 C is achieved in LiFeO_2_ up to 30 cycles. However, surprisingly, the specific capacity first decreases after 30 cycles down to ~ 350 mAh g^−1^ at 60 cycles, then gradually increases to 450 mAh g^−1^ at 120 cycles and then decreases again with capacity ~ 350 mAh g^−1^ at 140 cycles. The details of this capacity variation are not known. A possible reason is the fluctuation of the Li occupancy in the octahedra site. Therefore, if one can use a proper chemical species to sit on the Fe-site and limits the fluctuation of Li-ion occupancy, it might enhance the material structural stability and subsequently improve the battery stability.

On the other hand, it has been demonstrated that the anatase titanium dioxide (titania, TiO_2_) based mesoporous materials as anode^26^ in LIBs exhibit a specific capacity of 260 mAh g^−1^ at a current density of 10 A g^−1^. The lithium titanate compound with a stoichiometry of Li_4_Ti_5_O_12_ has become a candidate anode material for fast charging LIBs^27,28^_._ This material is also of substantial commercial interest. Therefore, it would be interesting to investigate the potential of combining TiO_2_ with Fe_3_O_4_ as a new design for the anode in LIBs. Xue et al*.* reported to successfully synthesize the Fe_3_O_4_ doped-TiO_2_ superparticles via a colloidal co-assembly route followed by calcination, and the sample exhibits highly enhanced electrochemical properties^29^. However, the report did not carry out a detailed structural analysis.

Theoretical study has been performed to accurately describe many properties for Li-ion batteries. Concerning electronic property, DFT is an appropriate tool to reach this task. Multi-scale computational methods in line with top-down modelling are performed also for Li-ion batteries. A detailed computational investigations on Li-ion battery materials have been reviewed by Shi et al*.*^30^. Furthermore, investigation on specific battery property have been booming by several data driven approaches^31–33^. Down the lane, optical band gaps of doped-ZnO films and doped-TiO_2_ photocatalysts, redox potentials for lithium-ion batteries, are predicted by using Gaussian process regression model by Zhang et al*.*^31–33^. Therefore, it is tempting to study the electronic structures of Ti doped Fe_3_O_4_ and the pristine Fe_3_O_4_ using DFT calculation, ML and thereby correlating the structural and electrochemical data in a reasonable style.

In this paper, we report the electrochemical and battery performance of Ti doped Fe_3_O_4_ and the pristine Fe_3_O_4_ that are prepared by the co-precipitation technique. It has become an emerging trend to use computational methods coupled with machine learning^34–36^ to study the effect of different dopants and prediction of their chemical properties. The synthesis of 0.2%-Ti doped Fe_3_O_4_ is then designed by using a machine-learning method to avoid the impurity formation as demonstrated in the phase diagram of Fe–Ti–O (Figure S1). The structural, morphological and electrochemical studies are performed to examine the stability of the material as an anode in LIBs. High cycling stability is achieved upon Ti-doping. The capacity reaches 450 mAh g^−1^ after 10 cycles and maintains at the same value up to 100 cycles, demonstrating that the Ti-doping on the Fe site is a workable strategy for better battery performance. Moreover, post-mortem analyses of X-ray diffraction (XRD), scanning electron microscopy (SEM) and electrochemical impedance spectroscopy (EIS) help to better understand the origin of high capacity retention.

## Results and discussion

**Figure 1:**
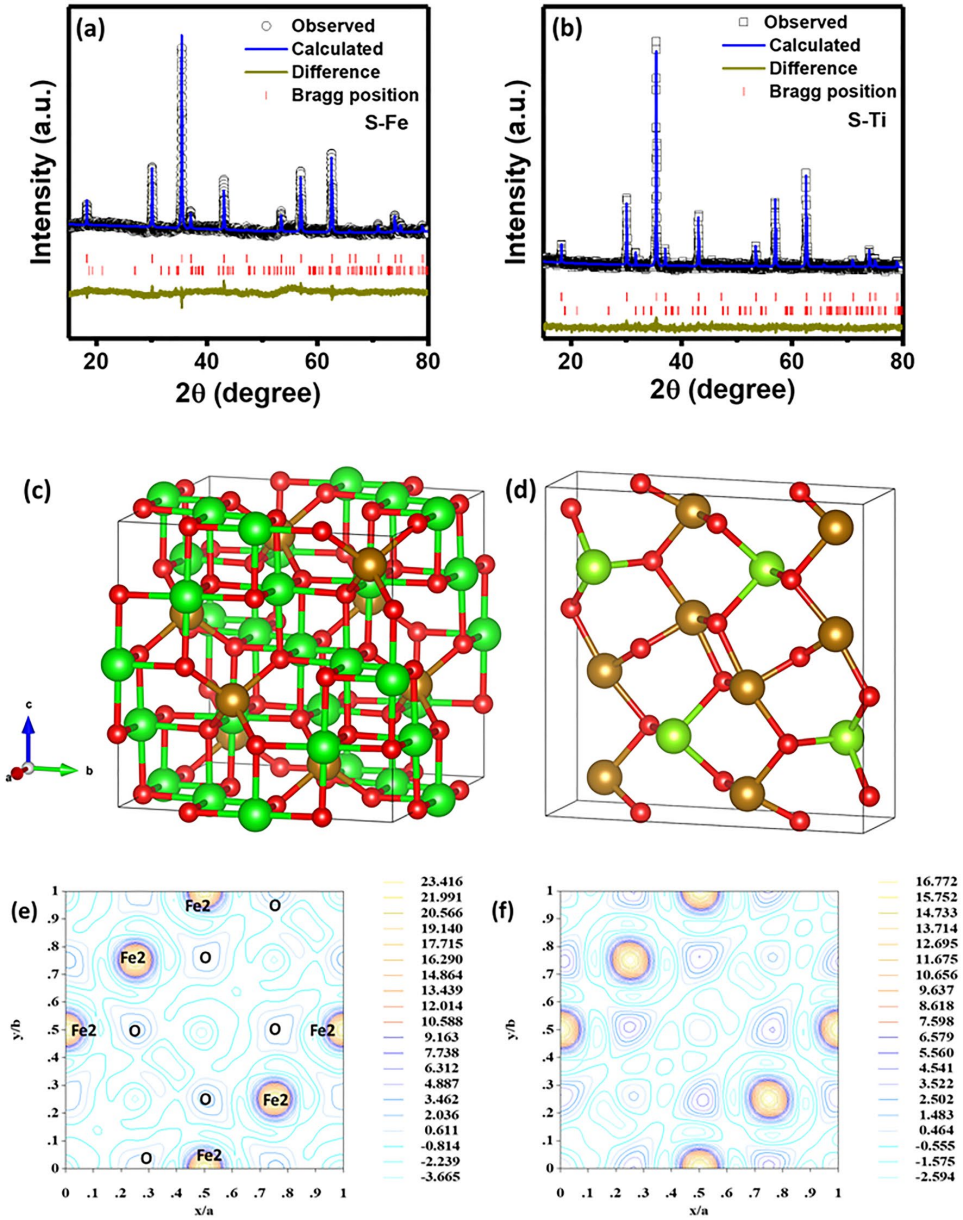
Rietveld refinement of the XRD patterns for (**a**) S–Fe and (**b**) S–Ti samples at room temperature. The top and bottom Bragg positions correspond to F d − 3 m and P b c m respectively in both panels. Crystal structures of S–Ti sample considering ionic radii of the atoms for representation in (**c**) Cubic and (**d**) Orthorhombic phases. Green and brown color correspond to two different sites of Fe having + 2 and + 3 oxidation states respectively in both structures. Electron density distribution difference for (**e**) S–Fe and (**f**) S–Ti along *ab* plane considering cubic spinel phase only. The atomic nomenclature is removed for better visibility in (**f**).

For the sake of simplicity, we name S–Fe and S–Ti for Fe_3_O_4_ and Ti-doped Fe_3_O_4_ nanoparticles, respectively. The X-ray diffraction (XRD) patterns of S–Fe and S–Ti show excellent crystallinity (Fig. 1a,b). We observed in the XRD a small peak at 2*θ* of 31.8° for both S–Fe and S–Ti samples as shown in Fig. 1a,b suggesting the existence of a secondary phase. By calculating the ratio of the peak intensity at 31.8° to that of 35.4° (the main peak for cubic phase) for both samples, the results are ~ 0.7% and 5.5% for S–Fe and S–Ti samples, respectively. We have followed the regular refinement strategies, by normalizing the atomic co-ordinates and the site occupancies as a pre-processing step, to perform quantitative phase analysis using Rietveld refinement technique. To perform the quantitative phase analyses we have considered the peak intensities, structure factors as well as lattice information. The Rietveld refinements of the XRD patterns for both S–Fe and S–Ti confirm the presence of major cubic (F d − 3 m) and minor orthorhombic (P b c m) phases in the samples. The refinement model for cubic structure with origin at − 3 m is followed from the work by Wechsler et al*.*^37^. The Bragg peak around 31.8° in 2*θ* is identified as the characteristic of the orthorhombic phase. The sintering of the samples in a vacuum and sealed atmosphere helps to form the high-pressure orthorhombic phase^38^ for both samples. Typical crystal structures of S–Ti are shown in Fig. 1c,d, exhibiting both cubic and orthorhombic crystal structures, respectively. The atomic coordinates for all the samples according to the different phases are presented in Table 1. The Rietveld refinements show that Fe has both + 3/ + 2 valence states and the orthorhombic phase contains fractional coordinates of both Fe and O atoms. Based on the X-ray diffraction results, both the cell parameters and cell volumes increase with Ti doping (see Table 1). We have calculated the electron density distribution considering only the major cubic phase. As observed from Fig. 1e,f, a relatively high positive electron density difference is observed for the Fe2 site for both samples, whereas the net density difference decreases with Ti doping, justifying Ti to be on the Fe2 site (Wyckoff position of 16d, octahedral units). Interestingly, the covalent bonding nature decreases remarkably due to Ti doping. Ionic bonding between Fe and O as well as Fe and Fe is noted for both samples. O gets relatively positive density with Ti doping as observed from Fig. 1f. These results are consistent with the identified Fe1 and Fe2 sites having + 3 and + 2 oxidation states of Fe, respectively. This is further verified by Bader charge analysis as desccribed later.

**Table 1:** Rietveld refinement parameters as obtained for S–Fe and S–Ti samples at room temperature.

Sample name	S–Fe	S–Ti
Crystal system	Cubic	
Space group	F d − 3 m (No. 227)	
Lattice parameter	8.3979 Å	8.3991 Å
Cell volume	592.27 Å^3^	592.52 Å^3^
**Atomic coordinates**		
Fe1 (8a)		
x	0.125	0.125
Normalized Occupancy	0.042	0.042
Fe2 (16d)		
x	0.5	0.5
Normalized Occupancy	0.083	0.083
O (32e)		
x	0.2555	0.2555
Normalized Occupancy	0.167	0.167
Crystal system	Orthorhombic	
Space group	P b c m (No. 57)	
Lattice parameter	a = 2.8231 Å	a = 2.82020 Å
	b = 9.4779 Å	b = 9.4378 Å
	c = 9.2129 Å	c = 9.3820 Å
Cell volume	246.52 Å^3^	249.72 Å^3^
**Atomic coordinates**		
Fe A (4d)		
x	0.724	0.724
y	0.3757	0.3757
z	0.25	0.25
Normalized Occupancy	0.5	0.5
Fe B (8e)		
x	0.246	0.246
y	0.1107	0.1107
z	0.0879	0.0879
Normalized Occupancy	1	1
O 1 (4c)		
x	0.506	0.506
y	0.25	0.25
z	0	0
Normalized Occupancy	0.5	0.5
O2 (4d)		
x	0.18	0.18
y	.2447	.2447
z	.25	.25
Normalized Occupancy	.5	.5
O3 (8e)		
x	.296	.296
y	.4899	.4899
z	0.098	0.098
Normalized Occupancy	1	1

The surface elemental composition and oxidation states of S–Fe and S–Ti are analyzed using the X-ray photoemission spectroscopy (XPS). The results are displayed in Figure S2. The survey spectra (Figure S2(a)) contain mainly Fe, Ti, C and O in their respective curve. The deconvolution of spectra is performed to identify the spin–orbit doublets for the S–Ti sample. Figure S2(b) shows the high-resolution XPS spectrum of the Fe 2p regime, which is fitted with two spin–orbit doublets and a satellite peak, contributing characteristics of Fe^2+^ and Fe^3+^. The results are consistent with that reported elsewhere^39^. O 1s spectrum is also deconvoluted into two peaks at 531.17 eV and 534.03 eV (Figure S2(c)). The former peak is related to Fe–O species corresponding to Fe_3_O_4_ whereas the other peak due to the presence of residual oxygen containing groups (such as (O–H)) in the sample^40–44^. Furthermore, the deconvolution of the Ti 2p spectrum consists of a Ti^4+^ oxidation state with a satellite peak (Figure S2(d))^45^.

**Figure 2:**
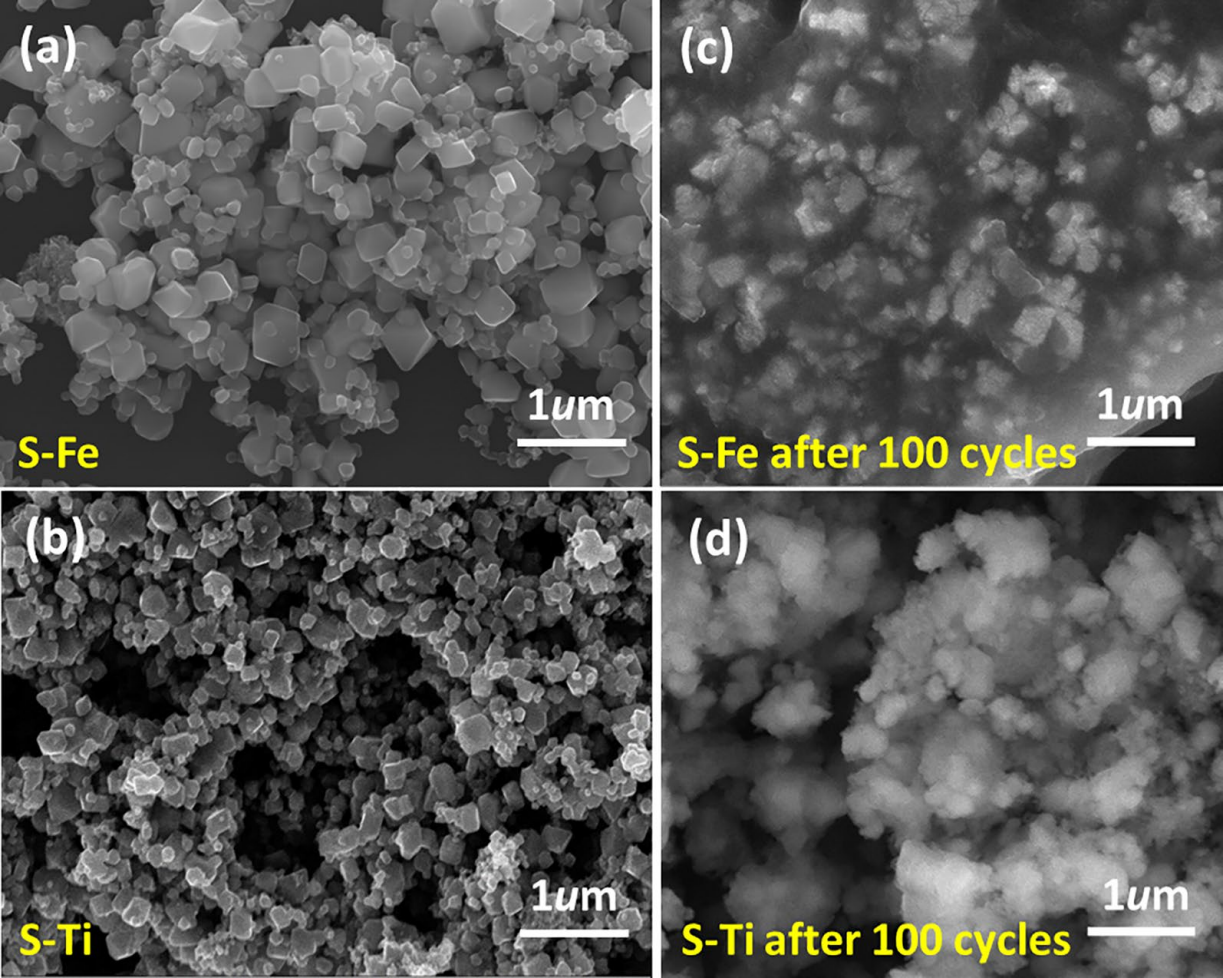
SEM micrographs for both (**a**) pristine S–Fe and (**b**) S–Ti; (**c**) and (**d**) correspond to after 100 cycles for (**c**) S–Fe and (**d**) S–Ti respectively.

The particle size and surface morphologies are well-controlled based on the scanning electron micrographs as shown in Fig. 2. The average size of Fe_3_O_4_ nanoparticles is about 257 nm which reduces to 176 nm after Ti doping (see Figure S3). For more meaningful interpretations we have added histograms of the particle size distribution for both S–Fe and S–Ti samples after 100 cycles (Figure S3). Particles become agglomerated in S–Fe sample after 100 cycles. As shown in Figure S3, the particle size increases from 176 to 250 nm for S–Ti after 100 cycles, whereas for S–Fe that decreases from 257 to 196 nm. As the particle size decreases exhibiting the broadening of XRD peaks, the capacity fading for S–Fe can be attributed to the pulverization related process (sometimes known as electrochemical milling). On the other hand, the increase in particle size due to prolonged cycling for S–Ti suggests proper ion transfer across the smooth-shaped and well dispersed particles (perfect Gaussian distribution in Figure S3(d)). Moreover, lattice parameter of S–Ti cubic phase increases as confirmed by the Rietveld refinement due to prolonged lithiation/delithiation processes. It is also observed the particles are separated for both S–Fe and S–Ti samples, as shown in Fig. 2a,b respectively. After cycling the particles are packed with a glue-like feature in S–Fe but not in S–Ti (Fig. 2c,d, respectively). The high morphological stability observed for S–Ti helps achieve high capacity retention, which will be discussed later. Similar morphological stability has been reported for the Fe_3_O_4_@C composite^19,46^.

**Figure 3:**
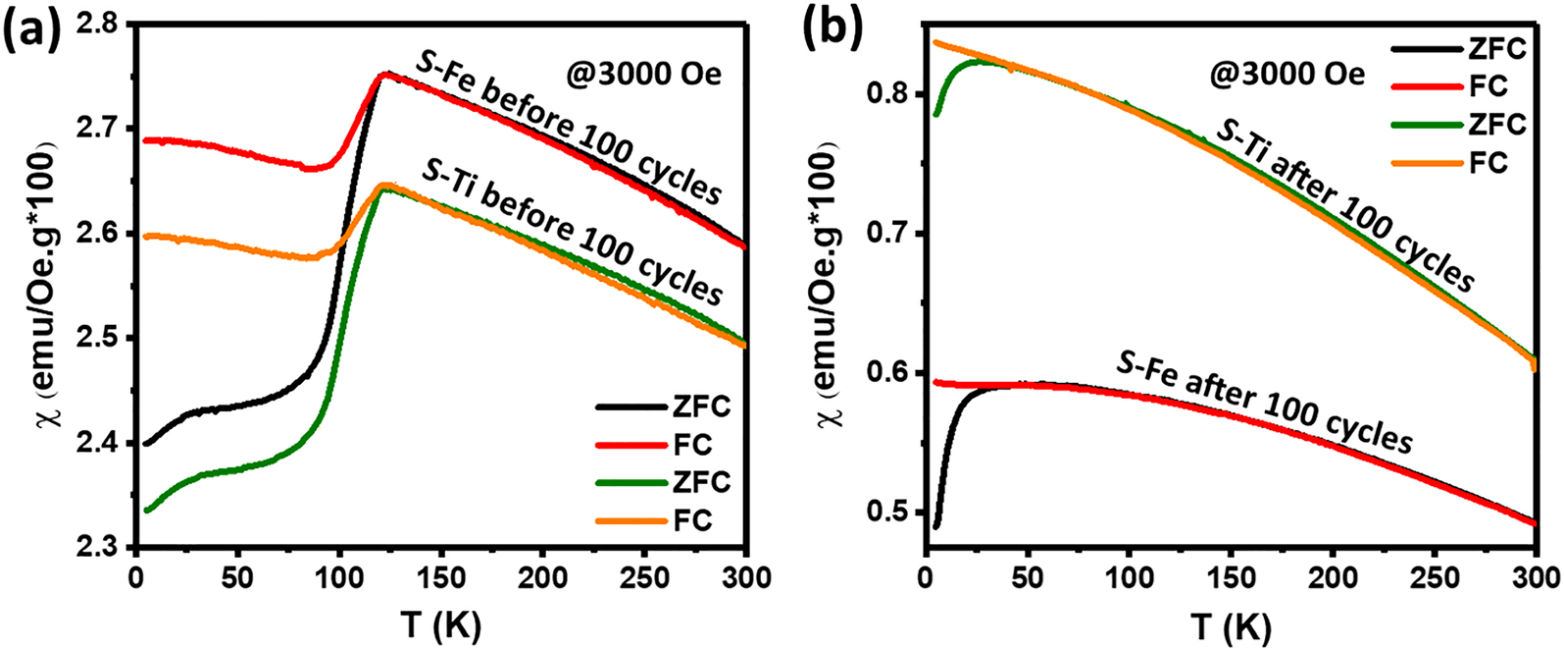
Susceptibility plots at FC and ZFC modes of S–Fe and S–Ti samples (**a**) before charging–discharging, and (**b**) after 100 cycles.

The temperature dependence of susceptibilities ($\chi =M/H$) measured in zero-field-cool (${\chi }_{ZFC}$) and field-cool (${\chi }_{FC}$) processes at 3000 Oe field for the samples before and after 100 charging-discharging cycles are shown in Fig. 3. Both curves in Fig. 3a show a diamagnetic signal at ~ 122 K, which is the signature of the well-known Verwey (metal–insulator) transition due to the ordering of Fe^2+^ and Fe^3+^^47^ in magnetite structure. A second anomaly with the onset at ~ 30 K, which is reported related to the growth of magnetic clusters^48^ is also observed in Fe_3_O_4_. It is rather surprising to observe the Verwey transition in both S–Fe and S–Ti samples is completely suppressed after 100 charging-discharging cycles as shown in Fig. 3b. The exact origin of this effect, which is most likely due to the incorporation of Li-ion that disrupts the charge-ordering in Fe_3_O_4_, is currently under further investigation. On the other hand, the low-temperature anomaly seems not much affected in both S–Fe and S–Ti samples after 100 cycles (Fig. 3b) except that the susceptibility value is substantially reduced. The estimated magnetic moments of the samples by first-principles calculations are 4.25 µ_B_ and 4.14 µ_B_ per formula unit for S–Fe and S–Ti considering Ti is doped in Fe2 site. The results are consistent with the estimated effective magnetic moments for all samples, using the Curie–Weiss relation based on the linear-regime in χ vs. 1/T curve (Figure S4). The measured moments are reduced to 1.22 µB, and 1.17 µ_B_ for S–Fe, S–Ti after 100 cycles respectively.

**Figure 4:**
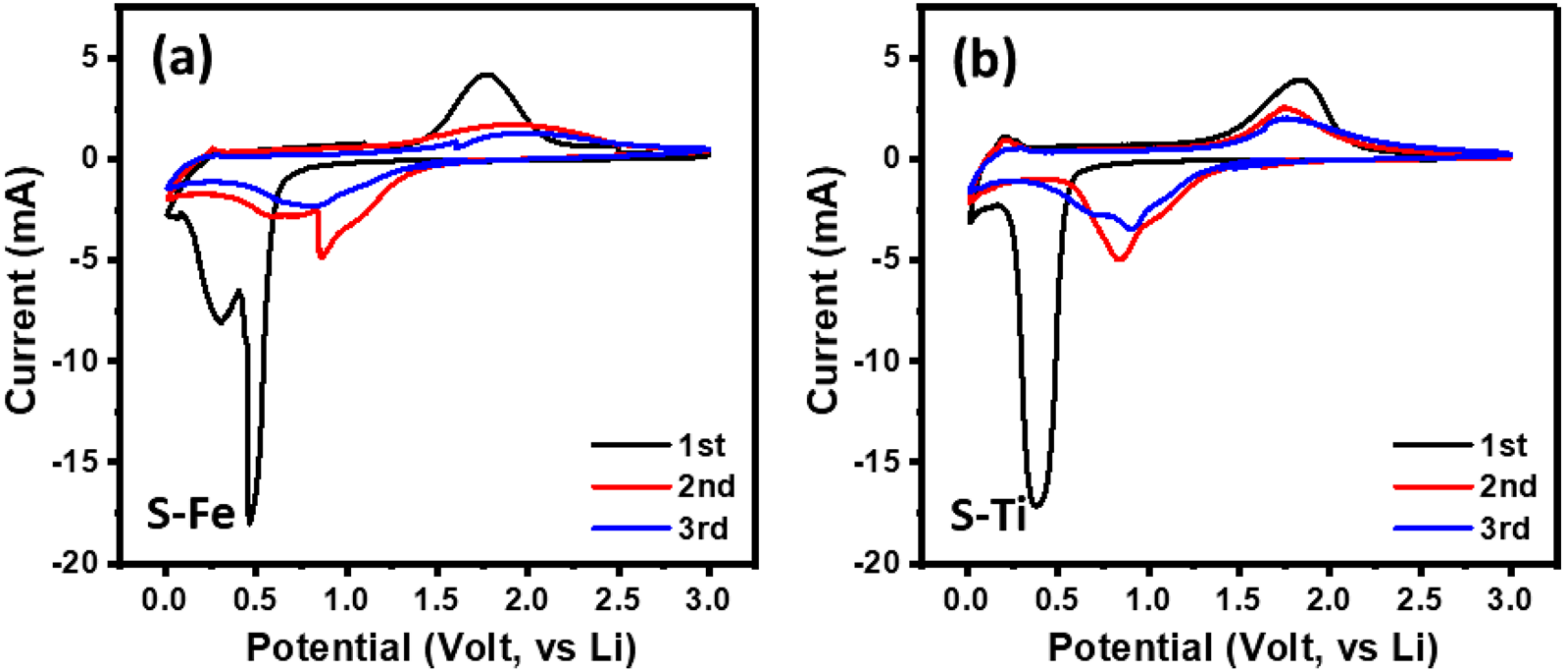
CV curves for (**a**) S–Fe and (**b**) S–Ti Li cells after several cycles.

**Figure 5:**
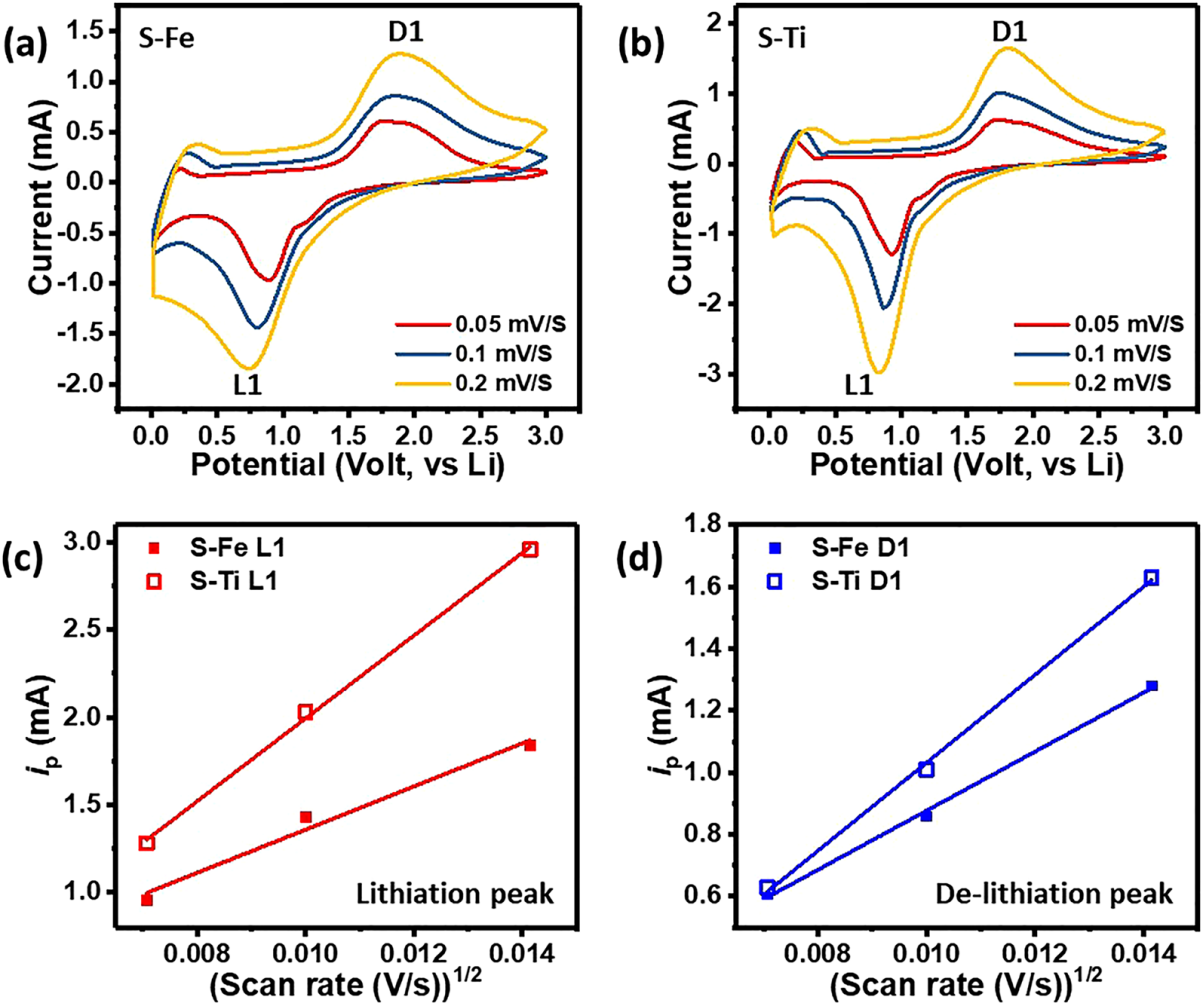
CV curves of (**a**) S–Fe and (**b**) S–Ti anode based batteries at different scan rates of 0.05, 0.1 and 0.2 mV/s between 0.01 V to 3 V. Dependence of reduction and oxidation peak currents (*I*) on the square rate for (**c**) S–Fe and (**d**) S–Ti, respectively.

Figure 4a and b show the cyclic voltammetry curves for both S–Fe and S–Ti samples using LiPF_6_ as an electrolyte in coin-type cells. Cyclic voltammetry (CV) is used to obtain the apparent chemical diffusion coefficient of lithium ions. Figure 5a,b show the CV curves of S–Fe and S–Ti during the 5th cycle at different scan rates of 0.05, 0.1 and 0.2 mV s^−1^ between 3.0 and 0.01 V. The data show that the heights of the lithiation and de-lithiation peaks increase with increasing potential scan rate. As suggested by Rui et al*.*^49^, at a high scan rate, the redox peaks may become difficult to distinguish, therefore, the CV curves at three low scan rates were conducted. As shown in Fig. 5c,d, each redox peak current (*i*_p_) shows a linear relationship with the square root of scan rate (*ν*^1/2^), which is expected for the diffusion-limited lithiation/de-lithiation processes of Li-ion. We then apply the classical Randles-Sevchik equation to study the semi-infinite diffusion of Li^+^ into S–Fe and S–Ti anode. The following equation is derived from the adsorption process theory at the metal/solution interface^50–52^:

1 $$I\mathrm{p}={{(2.69\times {10}^{5})n}^{3/2}SD}_{{Li}^{+}}^{1/2}{C}_{Li}^{*}{v}^{1/2}$$

where *I*_p_ is the peak current (A), *n* is the charge-transfer number, *S* is the contact area between anode and electrolyte (1.54 cm^2^), D_Li+_ is the chemical diffusion coefficient (cm^2^ s^−1^), *C*_Li_ is the concentration of lithium ions in the anode material and *ν* is the potential scan rate (V s^−1^). Based on Eq. (1) and the slope values from Fig. 5c,d, the apparent diffusion coefficients for the peaks of L1 and D1 are calculated, and are listed in Table 2. The D_Li+_ values of S–Ti are higher than those of S–Fe for both lithiation and de-lithiation processes, indicating that the enhancement of Li^+^ ion diffusion kinetics by Ti-doping. This stabilizes the electronic structure and assists electron transfer as revealed by density of states (DOS) calculation as well as the point defect approach (Eq. (4)).

**Table 2:** The apparent diffusion coefficients of Li + in S–Fe and S–Ti anode calculated from CV.

Lithiation/De-lithiation peak	S–Fe L1	S–Ti L1	S–Fe D1	S–Ti D1
D_Li+_ (cm^2^/s)	4.32 × 10^–8^	8.28 × 10^–8^	3.34 × 10^–8^	4.97 × 10^–8^

**Figure 6:**
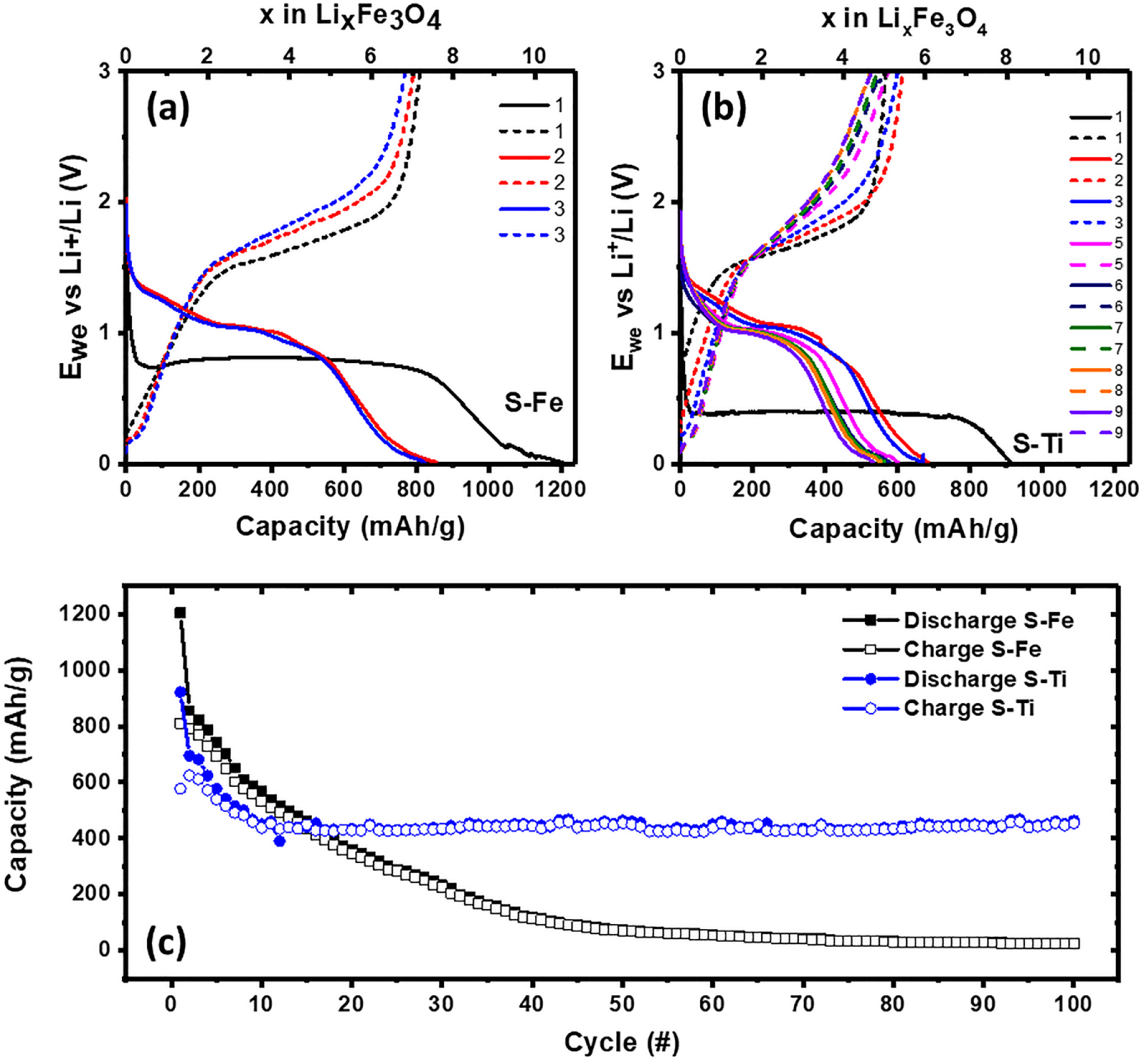
Voltage vs. capacity curves for (**a**) S–Fe and (**b**) S–Ti LIBs at different cycles at 0.1 C rate. (**c**) Cycling performance of both S–Fe and S–Ti LIBs at 0.1 C rate.

Figure 4 shows that the first cycles for both electrodes are different from that of their subsequent cycles. The origin for the observed difference is of solid electrolyte interphase (SEI) nature. As scanned from an open circuit potential (~ 1.65 V), a sharp peak at 0.5 V for S–Fe during discharging can be attributed to the formation of SEI layers^22^ as well as the reduction of Fe^3+^ and Fe^2+^ to Fe^0^^53^. Specifically, the transition from Fe_3_O_4_ to Li_x_Fe_3_O_4_ is made by assuming the insertion of almost 11 Li atoms per formula unit with a first discharge capacity of 1240 mAh g^−1^^54,55^. Although it is reported^55^ that upon de-lithiation about 8 Li atoms per formula unit could produce an initial charging capacity of 922 mAh g^−1^, our result indicates 7 Li atoms per formula unit with a charging capacity of 809 mAh g^−1^ (see Fig. 6a). Results suggested that the shoulder peak around 0.25 V is due to electrolyte decomposition^56^. During the first charging cycle the peak around 1.85 V can be due to the oxidation of Fe^0^ to Fe^3+^ with the following reversible oxidation reaction^57^:

2 $${3\mathrm{Fe}}^{0}+4{\mathrm{Li}}_{2}\mathrm{O}\leftrightarrow {\mathrm{Fe}}_{3}{\mathrm{O}}_{4}+{8\mathrm{Li}}^{+}+{8e}^{-}$$

Both the lithiation and de-lithiation peaks gradually decrease with increasing cycles suggesting the phase transition of the active material due to repetitive lithiation and de-lithiation processes. In the subsequent charging cycles the oxidation of Fe^0^ to Fe^2+^ and Fe^3+^ are associated in overlapping and broad peaks for Fe_3_O_4_ (Fig. 4a). Additionally, during 2nd discharge cycle, a new lithiation peak (~ 0.9 V) could follow the initial reduction reaction mechanism (Eq. (2))^57^. Eventually, this peak vanishes from 3rd discharge cycle onwards. Overall, we propose that during CV measurements, variation in SEI layer, decomposition of electrolyte, as well as several channel reactions of ion transport, result in the unstable battery performance^58^.

For the S–Ti sample from 2nd cycle onwards a minor peak around 1.2 V could be associated with the initial lithiation of Fe_3_O_4_ to form Li_2_Fe_3_O_4_ (Fig. 6a), which was identified by *in-situ* Mössbauer spectroscopy ^59^ and the reaction can be described by Eq. (3):

3 $${\mathrm{Li}}_{2}{\mathrm{Fe}}_{3}{\mathrm{O}}_{4}+{6\mathrm{Li}}^{+}+{6e}^{-}\to 3{\mathrm{Fe}}^{0}+4{\mathrm{Li}}_{2}\mathrm{O}$$

and a tiny peak around 0.75 V could be due to Fe^3+^ and Fe^2+^ transition. The de-lithiation peak gradually shifts towards low potentials as cycle number increases, indicating the stability of the compound (Fig. 6b). Moreover, it is observed that the capacity retention for S–Fe and S–Ti is 7 and 5 Li atoms per formula unit bound processes up to 3 cycles respectively. The voltage profiles lead to multiple steps of litihiaton processes at different cycles till reaching Li_x_Fe_3_O_4_ and allow Li to move between both tetrahedral and octahedral sites of spinel S–Ti (see Figure S5 for the tetrahedral and octahedral arrangements). Up to 100 cycles, about 4 Li atoms per formula unit transfer in the lithiation process for S–Ti are noted (Figure S6). These unique and multi-step processes of lithiation are helpful for stable and reversible capacity retention in S–Ti. The evolution of several peaks during lithiation as well as different cycles is due to different morphological effects and charge transfer processes, which can be understood through the DFRT analyses (to be presented in a later section).

Figure 6a,b compare the typical voltage profiles of both types of anode materials. At first, a large amount of irreversible capacity in both anodes is observed after the first cycle. The first discharge capacities are 1200 and 911 mAh g^−1^, corresponding to 10.5 and 8 Li atoms intercalation per formula unit for S–Fe and S–Ti, respectively. The irreversible capacity is most likely due to the formation of the solid electrolyte interface^52^. The irreversible capacity loss is observed in every cycle (for both samples) but for S–Ti, the loss is substantially reduced. For instance, the charging and discharging capacity difference for the 2nd cycle is ~ 120 mAh g^−1^ and 30 mAh g^−1^ for S–Fe and S–Ti respectively, indicating the superior performance of S–Ti. Another interesting feature of the S–Ti anode is the charging capacity in each cycle is comparable to the discharge capacity in its previous cycle. For instance, the 1st charging capacity (561 mAh g^−1^) is close to the 2nd discharging capacity (570 mAh g^−1^), corresponding to almost 5 Li atoms per formula unit from previous charging that are intercalated in the next discharging reactions. Figure S6 shows that after 100 cycles, S–Fe exhibits almost negligible accumulation of Li atoms, whereas S–Ti retains about 4 Li atoms per formula unit. Moreover, the lithium intercalation in S–Ti attains a saturation of around 4.5 Li atoms per formula unit from 8 cycles onwards, which could be a stable phase for further cycling effects. As already mentioned that up to 100 cycles we observe 4 Li atoms per formula unit transfer for S–Ti. Thus, lithiation and de-lithiation processes induce Li to interstitial sites in iron oxide spinel structure and once attains stable interstitial sites (4.5 Li atoms) a stable capacity is achieved.

The cycling performances at different charging conditions for S–Fe and S–Ti samples are shown in Fig. 6c. The specific capacity decreases with increasing cycling processes for both samples but much-improved performance is observed for S–Ti. The capacity continuously drops from an initial capacity of ~ 1200 mAh g^−1^ to ~ 430 mAh g^−1^ after 45 cycles for S–Fe. On the other hand, stable cycling after 15 cycles with a capacity of 410 mAh g^−1^ is achieved for S–Ti. Despite the low content of titania, the improved performance can be attributed to high loading density and smaller particle sizes, which produce a larger specific area of the electrode–electrolyte assembly and this is maintained up to 100 cycles.

Another explanation of high capacity retention for S–Ti can be provided by considering point defect chemistry. Using Kröger-Vink notetion the doping of Ti^4+^ on Fe^2+^ site generates electrons as

4 $${\mathrm{Fe}}_{3}{\mathrm{O}}_{4}\stackrel{3{\mathrm{TiO}}_{2}}{\leftrightarrow }{3\mathrm{Ti}}_{\mathrm{Fe}}^{\cdot\cdot}+3{\mathrm{Ti}}_{\mathrm{Ti}}^{\times }+10{O}_{O}^{\times }+6{e}^{-}$$

**Figure 7:**
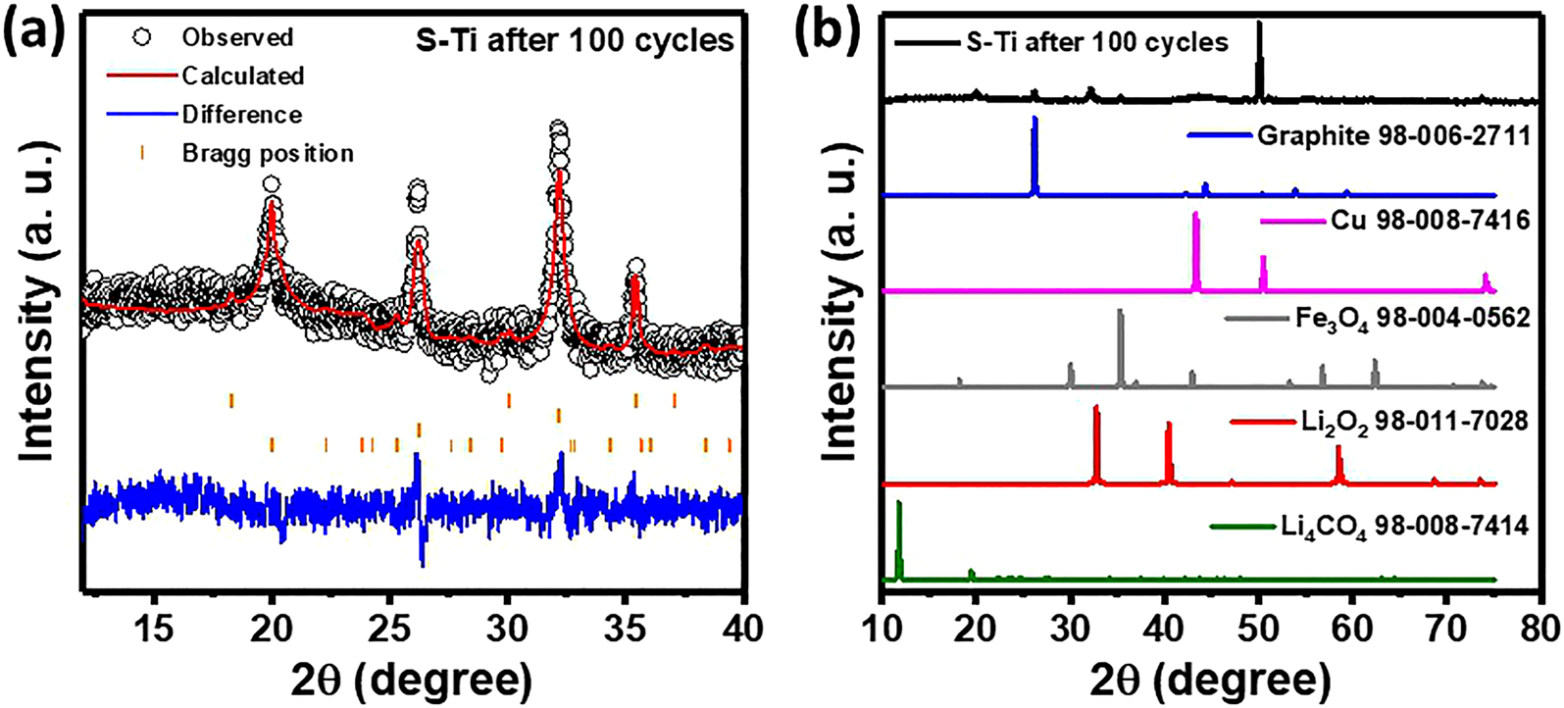
(**a**) Rietveld refinement of the XRD pattern for S–Ti sample after 100 cycles at room temperature. (**b**) Profile matching for S–Ti after 100 cycles. The ICSD database numbers that has been used to perform the matching are also shown.

From Eq. (4), ${Ti}_{Fe}^{\cdot\cdot}$ represents the net positive charge on the Fe site due to Ti. There is a lithiated phase with 5 and 6 Li atoms per formula unit, which is confirmed upon discharging with an increase in Li:Fe ratio (Fig. 6b). However, the electron density distribution difference implies high distribution of extra charges on both Fe and O atoms for S–Ti. Thus, there exists a lithiated phase (Li_4_Fe_3_O_4_) along with the parent phase (see Figs. 6 and 7) after 100 cycles. Consequently, the stability of crystal structures along with more ordered microstructure supports the higher stability in capacity for S–Ti.

The Rietveld refinement of the ex situ XRD of the S–Ti sample after 100 cycles confirms the presence of Li_2_O_2_, Fe_3_O_4_, Li_4_CO_4_ and carbon as shown in Fig. 7a. The refinement of the diffraction peak shifts, as shown in Figure S7 and Fig. 7b, indicates an increment in lattice constant from 8.3991 Å to 8.4064 Å, confirming the lithiated phase. Here we have considered only the cubic phase for S–Ti. The XRD pattern of S–Fe after 100 cycles contains broad humps between 15 to 50° in 2θ. The presence of carbon in either sample is due to the super P black carbon or due to the decomposition of electrolyte solution, which is mixed with EC, DMC. Based on Eq. (2), the final discharge product Li_2_O may react with the decomposed electrolytes that leads to Li_2_O_2_ species. Another possibility is that after the cycling experiments the EC, DMC react with the Li metal to form Li_2_O_2_ and Li_4_CO_4_ species. The slope around 0.5 V arises due to the first lithiation process for both electrodes during the 1st discharge process. In both discharge profiles from the 2nd cycle onwards a plateau around 1.36 V and 1.05 V implies a change in oxidation states Fe^0^ to Fe^2+^ and Fe^3+^, respectively. Similarly, a plateau around 1.5 V during charging is observed for both electrodes due to de-lithiation of Li_2_Fe_3_O_4_. The voltages for charging and discharging for S–Fe and S–Ti are 1.47 V and 0.8 V respectively, which justify different redox reactions associated with the electrodes. Overall, we have obtained half of the discharge plateau (0.4 V) for S–Ti than that of the S–Fe during the 1st discharge. Comparing both scanning electron micrographs after 100 cycles, S–Ti shows more granular structure whereas S–Fe shows low crystallinity and most of them look decomposed. In addition, the *ex-situ* XRD also suggests a better crystallinity for S–Ti in comparision with S–Fe along with a highly lithiated phase (4 Li atoms per formula unit) for the former.

To understand the improved cycling performance for S–Ti, we have performed density of states (DOS) calculation, and the results are shown in Figure S8. To describe the magnetic and electronic behavior and for accurate estimation of the electron correlation we have used the Dudarev et al.^60^ approach with an on-site Coulomb interaction U_eff_ = 4.0 eV. In all samples the valence band and conduction bands are dominated by O 2p and Fe 3d states. A band gap due to Fe 3d down spin profile is observed (Figure S8(b) for S–Ti), which is due to shifting of Fe 3d states around Fermi level. The occurence of the band gap is due to anionic redox mechanism^61^. However, with lithiation the hybridization of Fe 3d states becomes stronger and the band gap vanishes. A close look in Figure S9 provides us that upon lithiation O 2p has more states (1.22 states/eV) in S–Ti from 0.7 states/eV in S–Fe respectively (Figure S9). Furthermore, the calculated Bader charge for Fe (8a), Fe (16d) and O (32e) atoms are 1.647 e^−^, 1.544 e^−^ and − 1.183 e^−^ respectively for S–Fe, which shows a little variation upon Ti doping. For instance, the charges for Fe (8a), Fe (16d), Ti (16d) and O (32e) atoms are (1.557—1.6) e^−^, (1.166—1.617) e^−^, 1.889 e^−^ and − (1.065—1.111) e^−^ respectively. Considering Fe (8a) at + 3 valence state in S–Fe, after Ti doping the charge reduces justifying Fe^3+^ gets reduction which is opposite effect for O atoms. For lithiated S–Ti sample the charges for Fe (8a) and Fe (16d) are 1.710 e^−^, 1.511 e^−^ respectively which are higher than S–Ti, contrarily with Ti (16d) and O (32e) as 2.530 e^−^ and − (1.242—1.339) e^−^ respectively. The increasing net charge on Ti and O atoms is justified by the pDOS plot in Figure S9, as non zero spin down contribution is observed around Fermi level for both atoms. Overall, electron transfer assisted redox chemistry processes are preferred in S–Ti. This is also in accord with Eq. (4).

**Figure 8:**
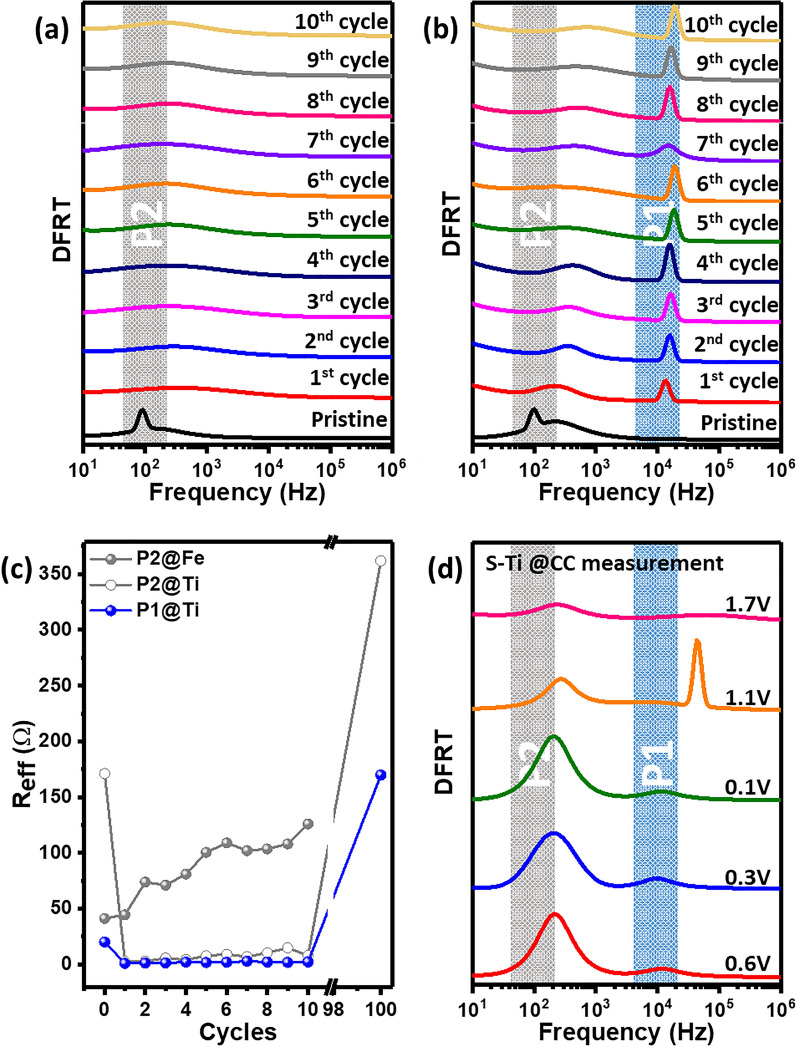
The DFRTs are shown in (**a**) S–Fe and (**b**) S–Ti after different cycle measurements. For description of the peaks (P1, and P2) the readers are referred to the text. (**c**) Variation of the effective resistances with cycles as obtained from peak P1 and P2 for both S–Fe and S–Ti samples. (**d**) DFRTs, as obtained for S–Ti sample at different potentials as achieved by constant current mode during 1st cycle of CV measurements.

Electrochemical impedance spectroscopy (EIS) is employed to study the ion transport phenomena in LIBs after each cycle measurement. As shown in Figure S10, the impedance responses from both samples look similar, having an Ohmic drop, along with a single and depressed semicircle at the high to intermediate frequencies (charge transfer processes) and a spike-like extension at lower frequencies (solid-state Li ion diffusion in electrode). The DFRTs are shown after different cycle measurements in Fig. 8a,b for S–Fe and S–Ti, respectively. Single semicircle may not corresponds to a single relaxation process as confirmed for S–Ti by the DFRTs (Fig. 8b). The impedance responses for both electrodes after 100 cycles show distinguished difference as displayed in Figure S10c.

To understand the observed results qualitatively, the impedance responses have been modelled by computing the distribution function of relaxation times^62^. The DFRTs consist of two peaks around 10^4^ Hz (denote as P1) and 10^2^ Hz (denote as P2) at different cycles for S–Ti, whereas only P2 is observed for S–Fe which vanishes after 100 cycles (Figure S11). To understand the origin of the peaks in the DFRTs, data are compared with those obtained for symmetric Li||Li coin cells at pristine and after the 1st cycle of CV measurements as shown in Figure S12. The results suggest that the peak (P2) originates from Li ion transport due to the Li metal and electrolyte interface, whereas the peak (P1) is due to the charge transfer processes across the S–Ti electrode and electrolyte solely, as it not observed in symmetric Li||Li cell (Figure S12). The effective resistances for both samples are shown in Fig. 8c. A large initial drop in the resistance is observed for S–Ti, which is due to the initial arrangement of ionic species and also due to the potential sweep during 1st cycle. The value for S–Ti is comparatively lower than that of S–Fe and only increases slightly by cycling, whereas the effective resistance for S–Fe continuously increases with cycling. The difference in ionic nature for S–Ti and S–Fe samples originates from their different relaxation mechanism. One can imagine that the Li-ion migration in S–Ti is more caged and isolated as partially evident from the isolated and reduced electron density difference for Fe and O atoms (see Fig. 1e,f) along the ab plane. As a result, the Li-ion migration becomes faster as more energy levels are available for ion migration. Thus, our investigation reveals that for maintaining high cycle stability, the Li ion transport process (P1) should be maintained along with regular charge transfer processes across the electrode/Li metal interfaces. The absence of these phenomena leads to capacity fading as that observed for S–Fe. This DFRT based analysis could be taken as a fingerprinting method to predict stability in discharge capacity of electrode material.

The above results suggest a more detailed study on the Li-ion migration during charging-discharging processes will provide valuable information to better understand the mechanism for improving cycling stability. The results of 1st cycle CV measurement, shown in Fig. 4, exhibits only a single reaction in both lithiation (discharging) and de-lithiation (charging) processes, which are related to the uptake and release of Li-ion. Therefore, we have carried out a series of EIS measurements on S–Ti sample at chosen potential values, which are followed by DFRT analysis to better understand the effect of lithiation and de-lithiation processes. The measured EIS patterns are shown in Figure S13 for constant current (CC) mode, and corresponding DFRTs are shown in Fig. 8d. Overall, we have observed single and depressed semicircles. However, the difference in shapes is noted for both P1 and P2 peaks. For instance, the localization of P2 around 10^2^ Hz irrespective of potential sweeps of discharging to charging, suggesting that the lithium ion transport across lithium metal to the electrolyte is a continuous process. Meanwhile, P1, which is already identified to originate from the S–Ti electrode and electrolyte interfacial process, depends on charging and discharging potentials. More specifically, there is two different position of P1 peak localization due to discharging and charging (Fig. 8d). This analysis confirms that delithiation and lithiation peaks not only depends on voltages but also frequency-dependent.

## Conclusion

We have presented a detailed study on the electrochemical performance of cubic phase Fe_3_O_4_ and Ti doped Fe_3_O_4_ nanoparticles. The incorporation of Ti increases the lattice parameter. A 0.2% Ti doping has improved the cycling performance of the Li-ion coin cells in terms of capacity, and stability up to at least 100 cycles and a record stable capacity of 450 mAh g^−1^ is achieved. Both chemical and microstructural stability as well as the highly lithiated phase for Ti doped Fe_3_O_4_ are the origins for the stability in capacity. Detailed analyses using distribution function of relaxation time (DFRT) based on EIS measurements indicate improvement in the interfacial charge transfer resistances as well as lithium ion transportation across electrode–electrolyte interface for Ti doped Fe_3_O_4_ help enhance the electrochemical performance. Overall, we have used ML model, DFT, defect chemistry approach, crystal structure and impedance spectroscopy coupled with DFRT analysis to explain the high capacity retention for Ti doped Fe_3_O_4_ anode. ML model successfully estimates the doping probability and constructs a phase diagram; DFT calculates the magnetic property and electronic structures which are verified by using experiments (magnetometry and electrochemical analysis); DFRT analysis finds relaxation processes which helps to explain the battery cyclability. The results and analysis strategies presented are universal and can be used to design other materials for better LIB applications.

## Experimental section

### Electrode

The reagents of analytic grade (FeCl_3_·6H_2_O, FeCl_2_·4H_2_O, and NaOH) were used as raw materials, and chemical grade hydrochloric acid was used as modifiers. Firstly, FeCl_3_·6H_2_O and FeCl_2_·4H_2_O with a molar proportion of 1:1 were dissolved in deionized water, here marked as solution “A”. Then NaOH solution (3 mol·L^−1^) was added into the solution “A” by using a micropipette under constant magnetic stirring for 15 min to obtain the Fe_3_O_4_ nanoparticles. Afterwards, hydrochloric acid was added into the suspensions for 1 h at room temperature to modify the Fe_3_O_4_ nanoparticles. The particles were magnetically separated and repeatedly washed with ethanol. Then the products were dried overnight in a vacuum chamber. After that, Fe_3_O_4_ nanoparticles were transferred into a quartz tube and sealed in a vacuum. The sealing quartz tube was heated at 723 K for 3 h in a furnace. To obtain Ti doped Fe_3_O_4_ nanoparticles, titanium isopropoxide with a molar ratio of 0.2% was added into solution “A” with other experimental conditions kept unchanged.

### Electrode preparation and cell assembly

The electrolyte solution was prepared by adding 1 mol·L^−1^ of LiPF_6_ in a mixture of ethylene carbonate (EC) and dimethyl carbonate (DMC) having a 1:1 volume ratio. A Celgard polypropylene membrane was used as a separator. The positive electrodes were prepared by mixing the active materials, graphite (Super P) and polyvinylidene fluoride (PVDF) in weight ratios of 80:10:10 respectively. Upon addition of *N*-Methyl-2-pyrrolidone (NMP), a black viscous slurry was obtained. The viscous paste was coated onto a copper foil using a doctor blade and dried in a vacuum for 24 h at 373 K. The CR2032-type coin cells were assembled in an argon-filled glove box. The electrochemical properties of the samples in CR2032-type coin cells were tested at room temperature with metallic lithium as the counter electrode.

### Characterization and modelling techniques

The crystal structures of the prepared samples were analyzed by the X-ray diffraction (XRD) technique using Philips X’Pert diffractometer equipped with a CuKα X-ray source (λ = 1.5406 Å). XRD measurements were collected in the 2*θ* range 10–80° and scan rate of 0.001° min^−1^. The Rietveld refinements of the XRD patterns were carried out in the FullProf package^63^. The microstructures of all the prepared samples were studied in a scanning electron microscope (SEM, JEOL-Japan, JXA-840A). X-ray photoelectron spectra (XPS) of the samples were recorded in an X-ray photoelectron spectrometer (ULVAC-PHI 5000 Versaprobe II) using Al Kα source of energy of 1486.7 eV in the high vacuum of pressure ≤ 6.7 × 10^–8^ Pa.

Magnetic measurements were carried out with an applied field of 3000 Oe using a fully automated magnetometer (MPMS-5S from Quantum Design) using an ultrasensitive Superconducting Quantum Interference Device (SQUID) within 4–300 K. The as-prepared powders were loaded into a small plastic vial, then placed in a holder and inserted into a helium Dewar flask of the apparatus. For the measurement of dc magnetization (M), the magnetic field was applied in both field-cooling (FC) and zero field-cooling (ZFC) protocols. In the case of FC, the magnetic field of 3000 Oe was applied to the samples at room temperature and measurements were performed during cooling, whereas in ZFC, the samples were cooled in a zero magnetic field, but a non-zero field was applied during the heating processes.

Electrochemical impedance spectroscopy (EIS) was performed for the CR2032-type coin cells using a PARSTAT MC 1000 electrochemistry workstation with an AC amplitude of 10 mV between 1 MHz to 0.01 Hz. The distribution function of relaxation times (DFRTs) was calculated by Impedance Spectroscopy Genetic Programming (ISGP) program^64–68^ using the impedance spectra. The area of each peak is calculated separately by the package and then multiplied by maximum (un-normalized) resistivity to find the corresponding resistance. To find the effective resistance (${R}_{eff}$) of each peak the resistance is divided by the total area of the DFRT. Presently, we have considered only the polarization behavior eliminating the capacitive diffusion occurring at low frequencies. Secondly, we have used Kramers-Krönig relations to validate the EIS data and as observed from Figure S14 the KK compatibility is maintained up to 0.1 Hz. Thus the DFRTs are considered up to 0.1 Hz as shown in the analysis.

Cyclic voltammetry was performed at different scan rates of 0.05, 0.10 and 0.20 mV s^−1^ at room temperature for the CR2032-type coin cells using the same workstation between 3 V to 0.01 V. The charge and discharge profiles were collected by galvanostatic cycling between 0.01 V and 3 V vs. Li^+^/Li, applying a constant current of 0.1 C rate at room temperature with a Think Power battery testing system. Additional, EIS measurements were performed at a 0.1 C rate and at different potentials to understand the effect of lithiation and de-lithiation phenomena during the first cycle of S–Ti battery. The additional EIS coupled with CV measurements analysis is further modified by a potential sweep of rate of 0.1 mV s^−1^ during the first cycle of S–Ti battery.

The post-mortem analyses containing XRD, SEM, EIS and SQUID measurements were carried out after 100 fully discharged cycles.

Theoretical calculations were performed in the Quantum Espresso^69,70^ distribution based on density-functional theory, plane wave basis sets and pseudopotentials to represent the ion–electron interactions. The electronic Kohn–Sham wave functions were expanded using a plane wave basis set, up to 825 Ry for kinetic energy cut-off for density with plane wave energy cutoff of 75 Ry. The Brillouin zone is sampled using a Γ-centered $4\times 4\times 4$ Monkhorst–Pack k–grid and the spin-polarized electronic structure calculations were performed using $6\times 6\times 6$ k-grid. The calculations were performed in 56-atoms supercells with an energy tolerance of 10^–8^ eV per unit cell and the force acting on the atoms are less than 0.01 eV Å^− 1^. A convergence test of different k-points is presented for Fe_3_O_4_ supercell (Figure S15) confirming $4\times 4\times 4$ k-grid is sufficient to achieve convergence in electronic structure calculation. The Bader charge analysis is performed by a method developed by Henkelman et al.^71^_._ In this method analysis of the electronic charge density in which a division of a molecular charge into non-overlapping atomic domains within a Bader volume is adopted. In this work, the phase diagram and substitutional probabilities (Table S1) of different cations are calculated using a machine-learning *pymatgen* code^72^.

## Supplementary Information

Supplementary Information.

## Data Availability

The data that support the findings of this study are available from the corresponding authors upon request. The datasets generated and/or analysed during the current study are available in the Crystallographic Open Database repository, [3000327, 3000328].
