# Supplementary material for: A study on Ti-doped Fe3O4 anode for Li ion battery using machine learning, electrochemical and distribution function of relaxation times (DFRTs) analyses
Source: Sci Rep. 2022 Mar 22;12:4851. doi: 10.1038/s41598-022-08584-4 (PMC8941007; doi:10.1038/s41598-022-08584-4)
Supplement: Supplementary file 1 — Supplementary Information. [file 41598_2022_8584_MOESM1_ESM.docx]

Supporting Information

**A study on Ti-doped Fe_3_O_4_ anode for Li ion battery using machine learning, electrochemical and distribution function of relaxation times (DFRTs) analyses**

Po-Wei Chi^a^, Tanmoy Paul^a*^, Yu-Hsuan Su^a, b^, Kai-Han Su^a, c^, Cherng-Yuh Su^c^, Phillip M. Wu^a,d^*, Sea-Fue Wang^d^ and Maw-Kuen Wu^a^

^a^Institute of Physics, Academia Sinica

128, Section 2, Academia Road, Taipei 11529, Taiwan.

^b^Department of Engineering and System Science, National Tsing Hua University

101, Section 2, Kuang-Fu Road, Hsinchu 300044, Taiwan

^c^Institute of Manufacturing Technology and Department of Mechanical Engineering, National Taipei University of Technology (TAIPEI TECH)

1, Section 3, Zhongxiao E. Road, Taipei 106, Taiwan.

^d^Department of Materials and Mineral Resources Engineering, National Taipei University of Technology (TAIPEI TECH)

1, Section 3, Zhongxiao E. Road, Taipei 106, Taiwan.

**Corsponding Author**

E-mail: paultanmoy00@gmail.com; pmwu@gate.sinica.edu.tw

We have tried to identify the most suitable n-type dopant at substitutional sites of Fe_3_O_4_. In this context, we found possible dopants with their different oxidation states using a machine-learning method to estimate the probability that one ion will substitute Fe. Based on the supporting excel file, Ti^4+^ has certain probability to form an alloy with Fe^2+^. Inspired by this ML method assistance we have designed a unique synthesis process to dope a small amount of Ti to Fe site. Furthermore, the phase diagram of Fe-Ti-O suggests that most of the compounds with Fe^3+/2+^ states doped with Ti^4+^ are unstable with energy above Hull > 0 eV constraining only a limited doping concentrations. Thus, we prepared only 0.2 % Ti-doping for our study.


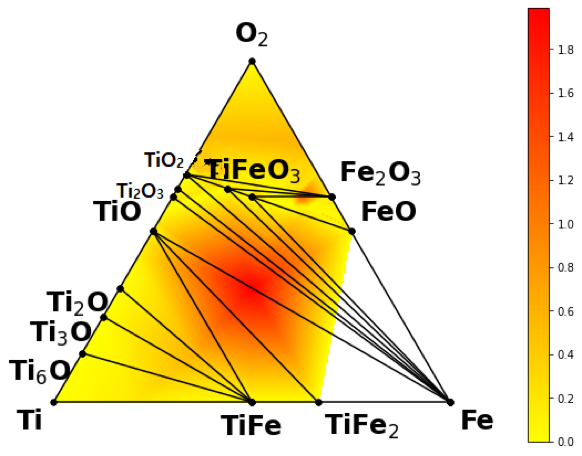


**Figure S1**. Contour plot of Fe-Ti-O phase diagram.


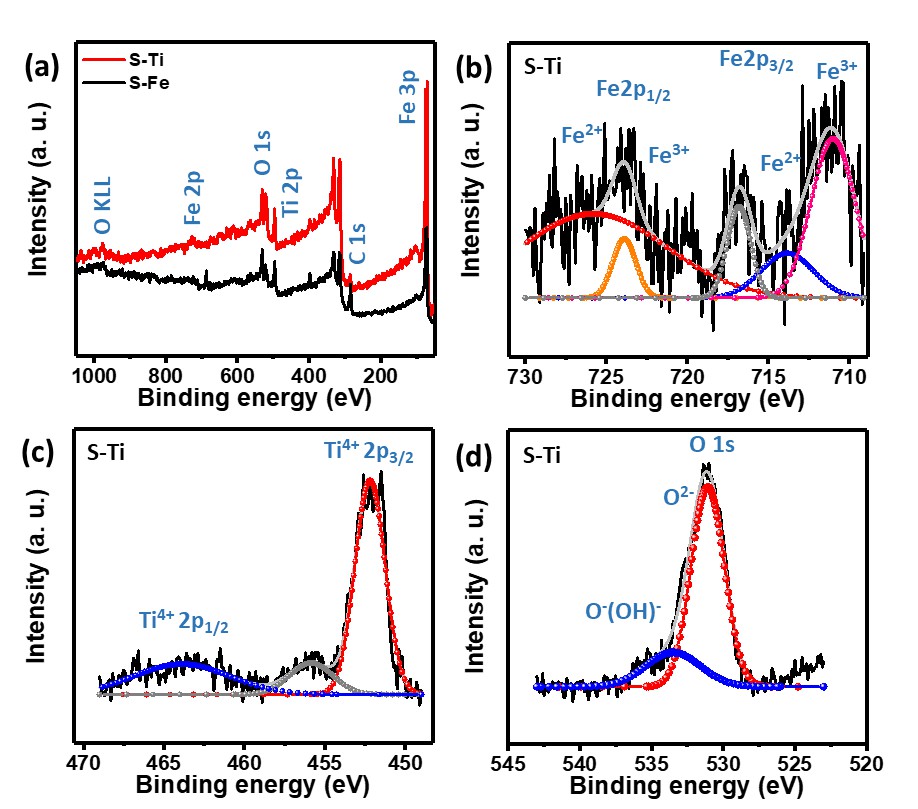


**Figure S2**. (a) Survey spectra for both S-Fe and S-Ti. Deconvoluted spectrum for (b) Fe (c) Ti and (d) O of S-Ti sample.


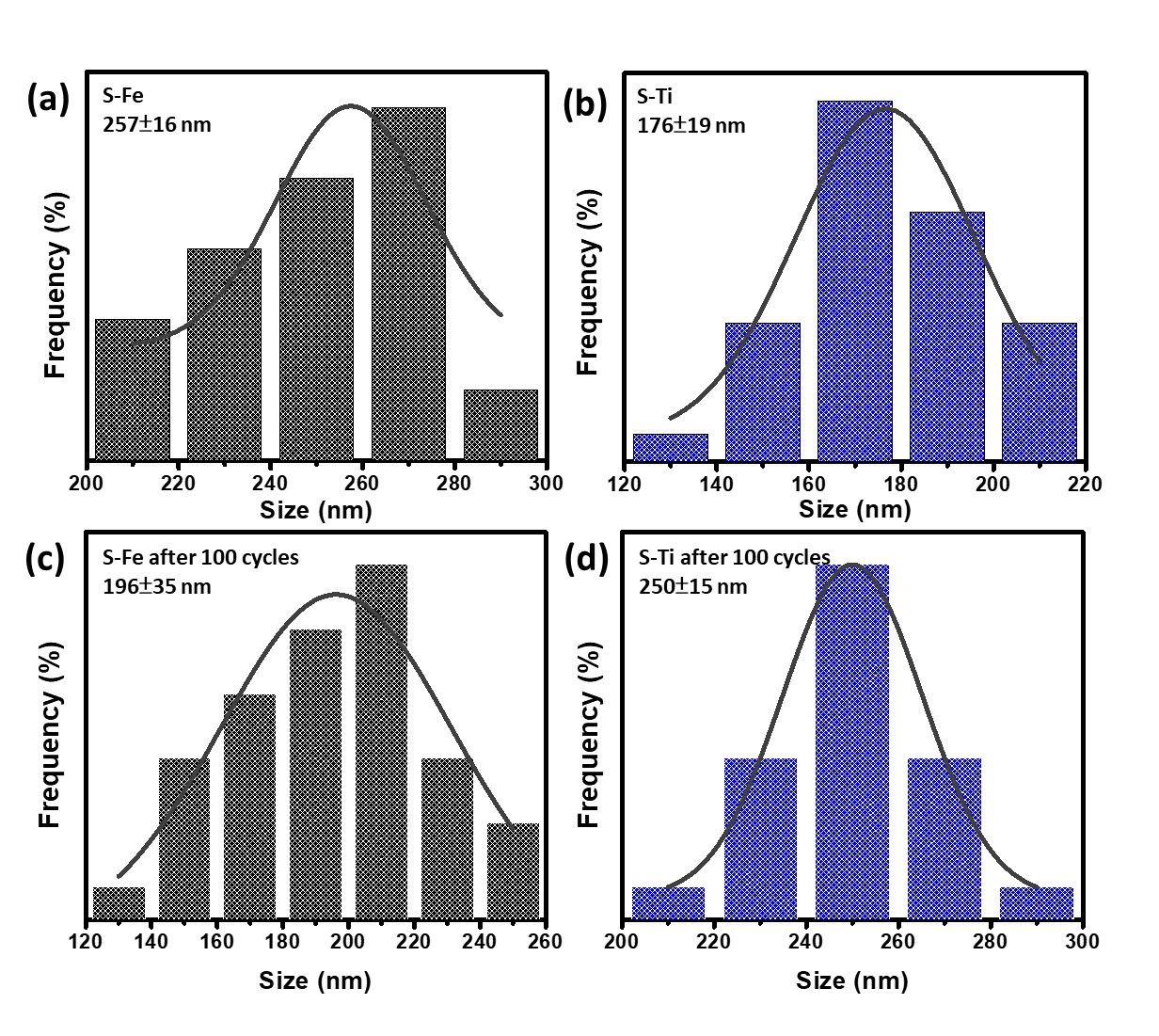


**Figure S3**. Particle size distribution for pristine (a) S-Fe (b) S-Ti and after 100 cycles (c) S-Fe (d) S-Ti.


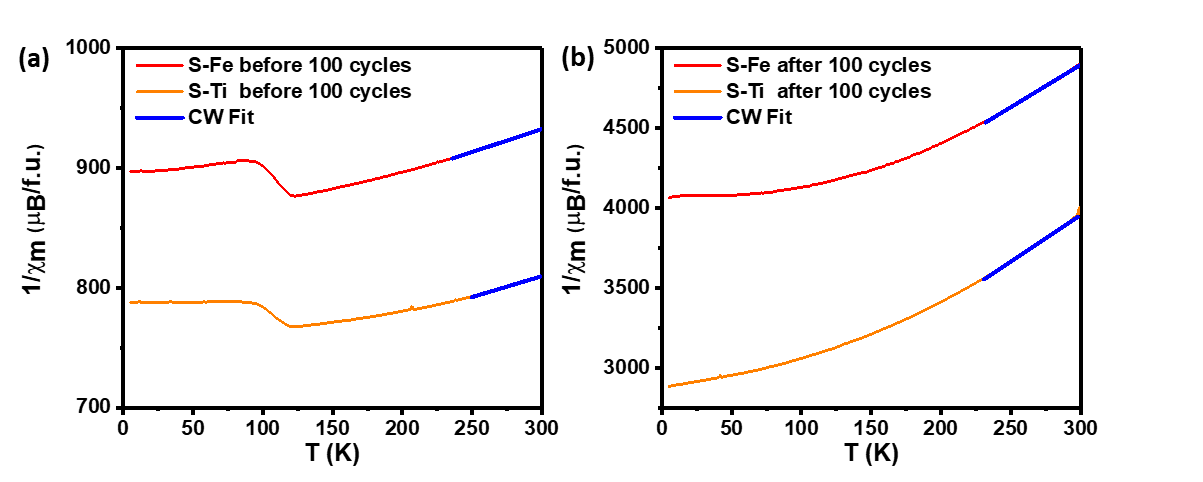


**Figure S4.** Temperature dependence of the inverse molar susceptibility (χ_m_) for both S-Fe and S-Ti (a) pristine samples, and (b) after 100 charging-discharging cycles. The blue lines show the Curie-Weiss law (CW) fitting.


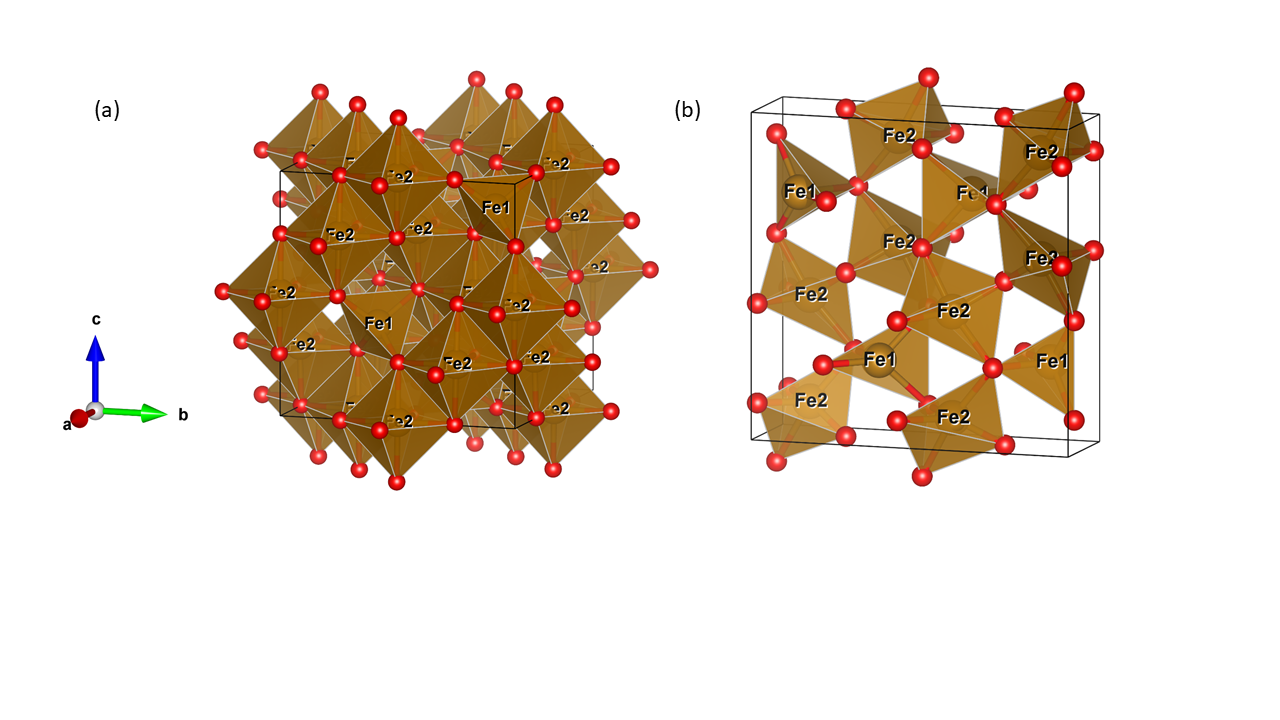


**Figure S5**. Polyhedral structure of S-Ti (a) cubic and (b) Orthorhombic with atoms as per atomic radii.


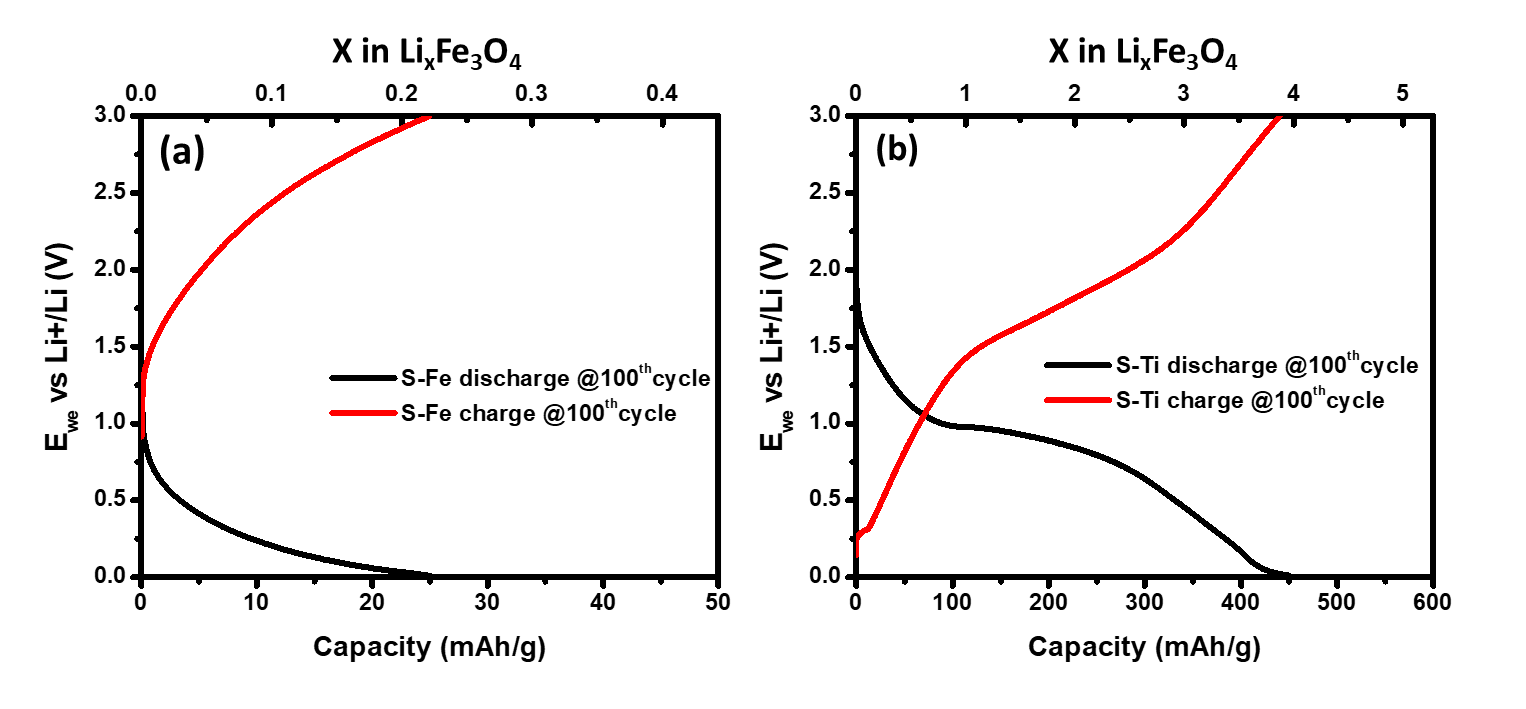


**Figure S6**. Voltage vs. capacity for (a) S-Fe and (b) S-Ti coin cells at 100^th^ cycle.


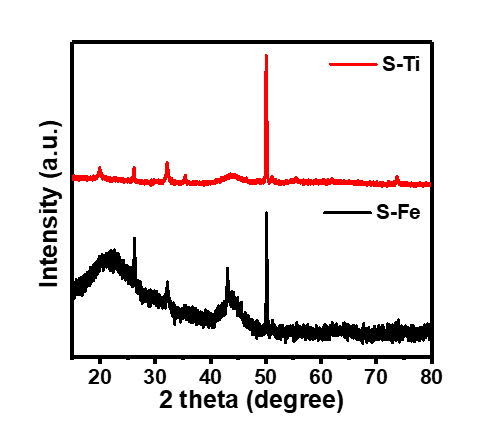


**Figure S7**. *Ex situ* XRD for both S-Fe and S-Ti after 100 cycles.


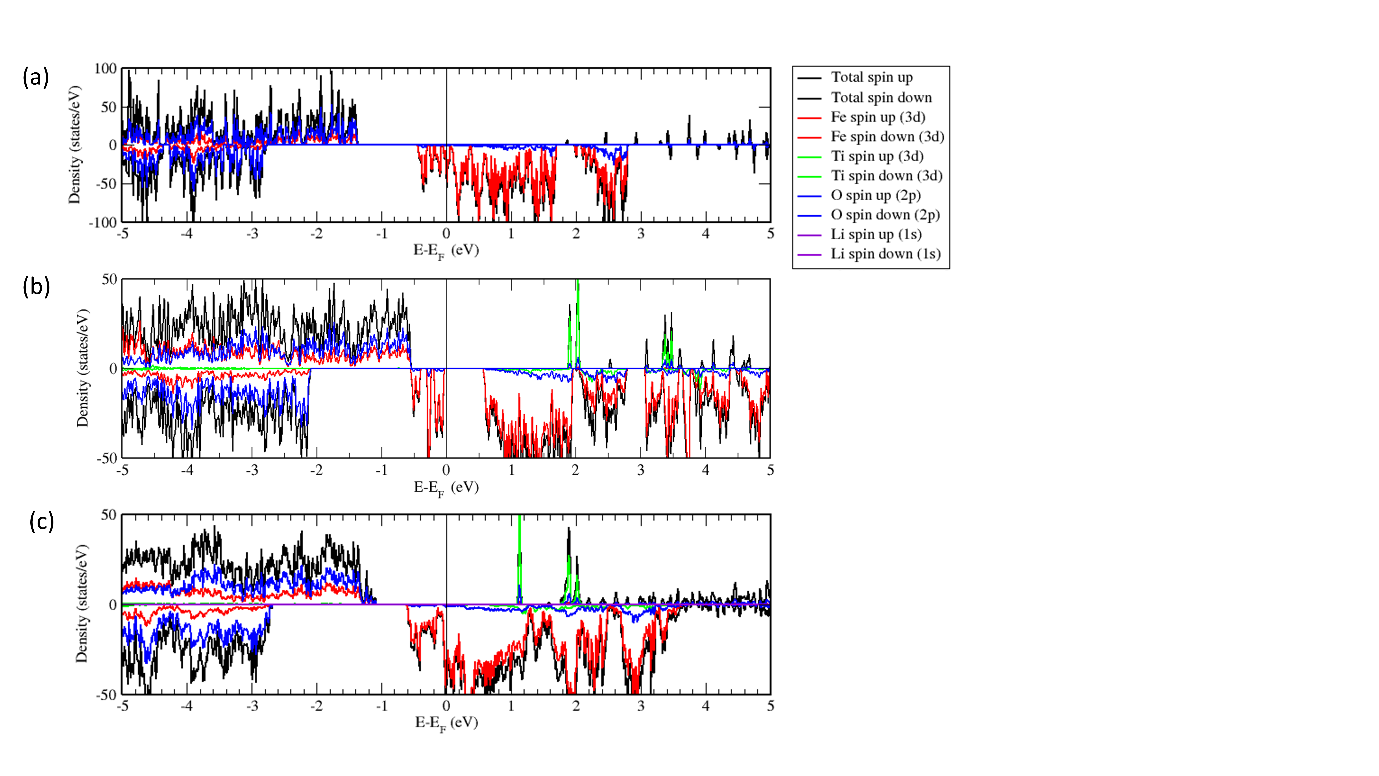


**Figure S8**. Spin polarized partial density of states for (a) S-Fe and (b) S-Ti and (c) Li_0.125_Ti_0.125_Fe_2.875_O_4_ samples respectively. The vertical blue indicates the Fermi Level (E_F_).


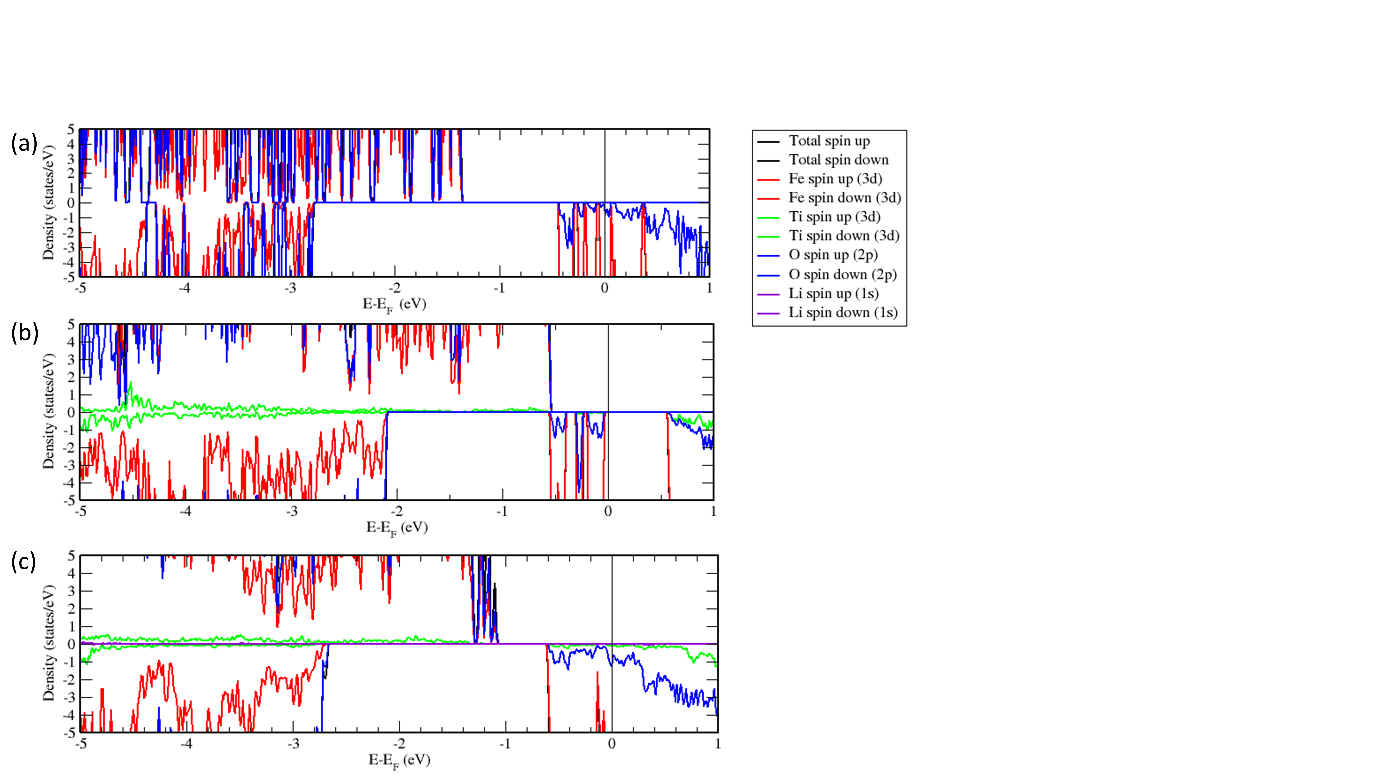


**Figure S9**. Spin polarized partial density of states for (a) S-Fe and (b) S-Ti and (c) Li_0.125_Ti_0.125_Fe_2.875_O_4_ samples respectively with zooming at the point of interest. The vertical blue indicates the Fermi Level (E_F_).


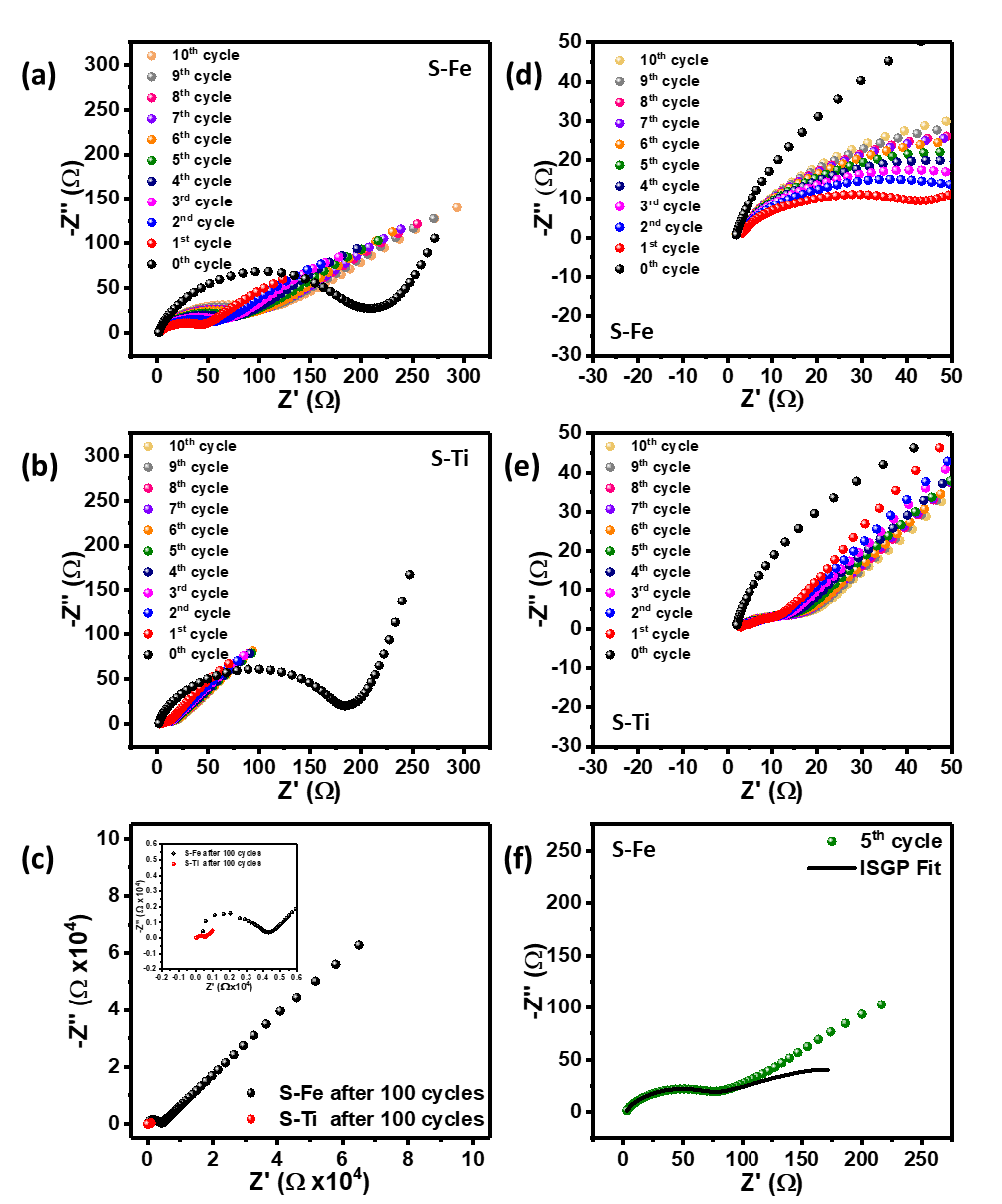


**Figure S10.** (a) and (b) show, respectively, the Nyquist plots for S-Fe and S-Ti after each CV measurement (up to 10 cycles); (d) and (e) are the enlarged part in low impednace region. (c) shows the Nyquist plot for both S-Fe and S-Ti after 100 cycles, and the inset displays the enlarged part of the low impedance region. (f) Typical ISGP fit is shown for S-Fe after 5^th^ cycle of CV measurement.


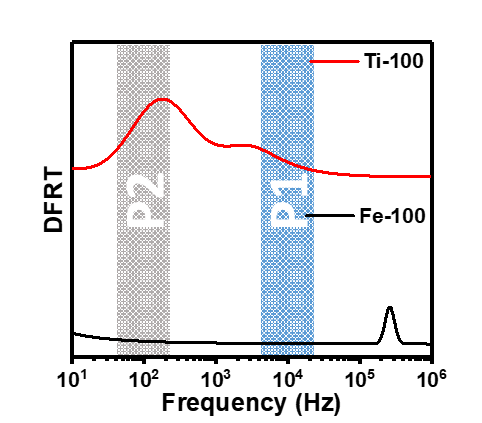


**Figure S11**. DFRTs obtained from Nyquist plots of Fig. S10(c) for both samples.


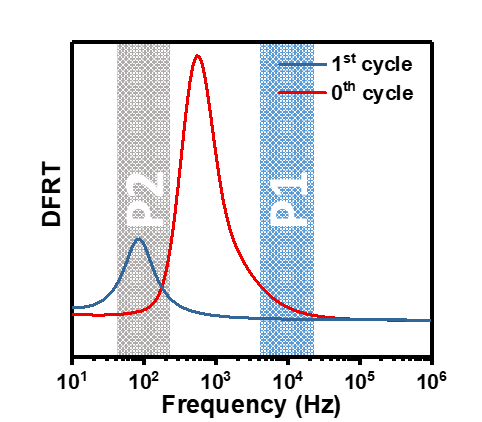


**Figure S12**. DFRTs before and after CV measurements for Li||Li symmetric cell.


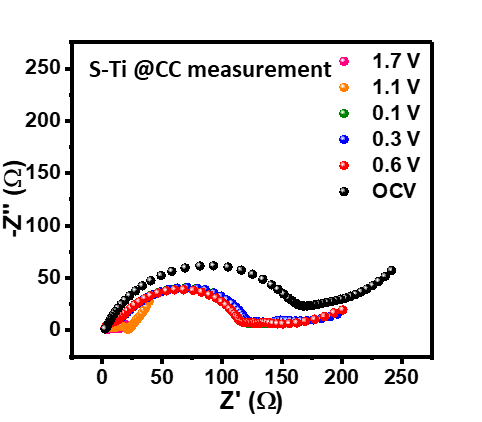


**Figure S13**. Nyquist plots of impedances obtained at different potentials by constant current mode for S-Ti electrode during 1^st^ CV cycle.


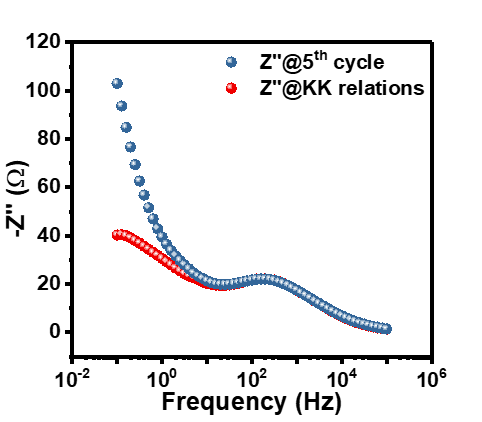


**Figure S14**. Frequency dependence of Zʺ for S-Fe after 5^th^ cycle of CV measurement along with that obtained from Kramers-Krönig relations.


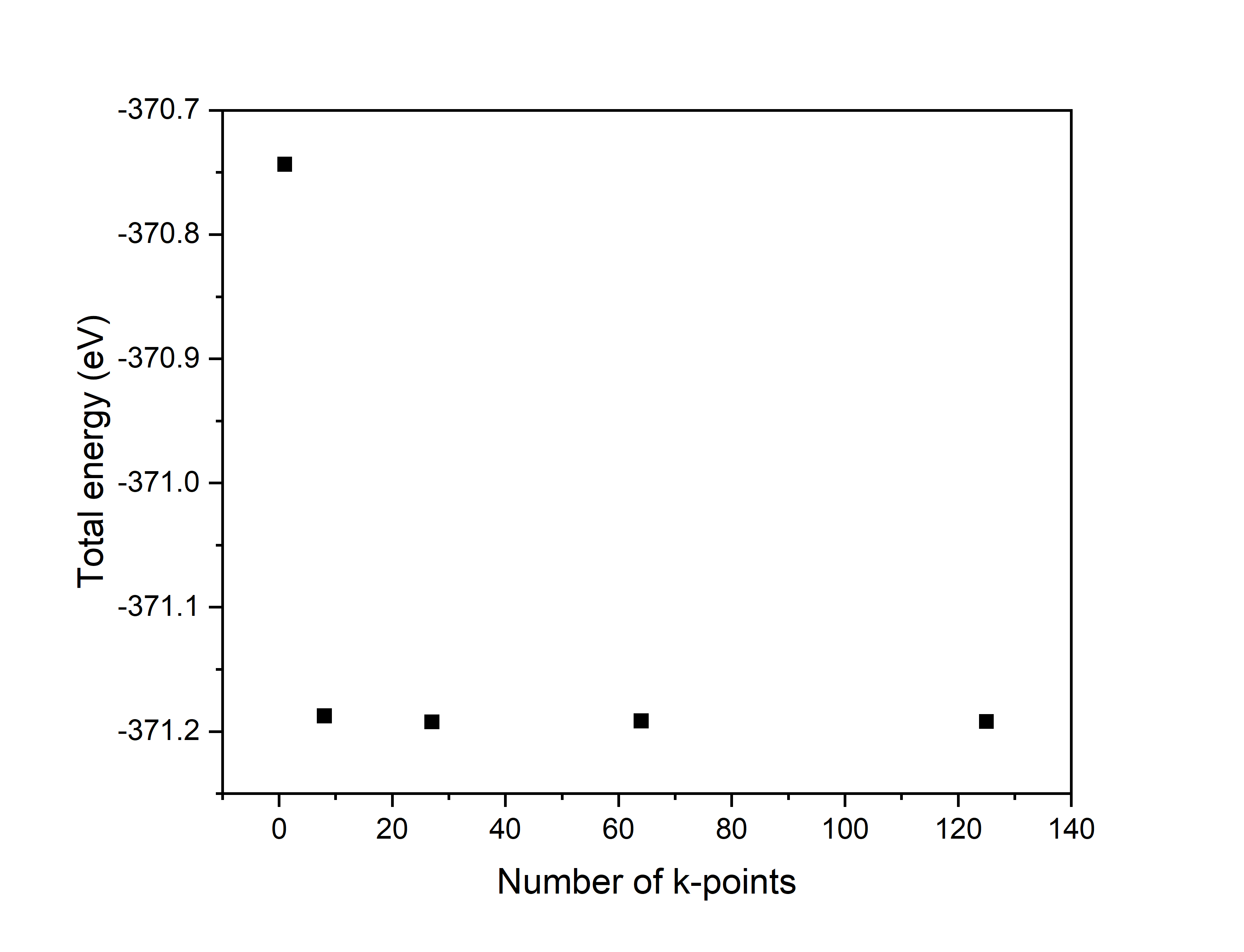


**Figure S15**. Convergence test with different k-points for Fe_3_O_4_ with 56 atoms supercell.

**Table S1.** Probability of different dopants that could be attached on the original atom site.

| Numbers | probability | dopant_species | original_species |
| --- | --- | --- | --- |
| 0 | 0.066927 | F- | O2- |
| 1 | 0.021697 | W6+ | Fe2+ |
| 2 | 0.021696 | Sn4+ | Fe2+ |
| 3 | 0.021023 | Cl- | O2- |
| 4 | 0.020978 | Cr3+ | Fe2+ |
| 5 | 0.016019 | Sb5+ | Fe3+ |
| 6 | 0.01447 | W6+ | Fe3+ |
| 7 | 0.013788 | Zr4+ | Fe3+ |
| 8 | 0.012621 | Ge4+ | Fe2+ |
| 9 | 0.012116 | Sb5+ | Fe2+ |
| 10 | 0.012115 | Mo6+ | Fe2+ |
| 11 | 0.010334 | V3+ | Fe2+ |
| 12 | 0.009856 | Se4+ | Fe3+ |
| 13 | 0.0096 | In3+ | Fe2+ |
| 14 | 0.009269 | Fe3+ | Fe2+ |
| 15 | 0.008909 | Mo6+ | Fe3+ |
| 16 | 0.00867 | Al3+ | Fe2+ |
| 17 | 0.008397 | Yb3+ | Fe2+ |
| 18 | 0.008397 | Ta5+ | Fe2+ |
| 19 | 0.008396 | Sc3+ | Fe2+ |
| 20 | 0.008122 | Si4+ | Fe2+ |
| 21 | 0.008121 | Mo3+ | Fe2+ |
| 22 | 0.007855 | Pt4+ | Fe2+ |
| 23 | 0.007855 | Lu3+ | Fe2+ |
| 24 | 0.007635 | Ti4+ | Fe3+ |
| 25 | 0.007635 | Ta5+ | Fe3+ |
| 26 | 0.007586 | Ru5+ | Fe2+ |
| 27 | 0.007586 | Ir5+ | Fe2+ |
| 28 | 0.007299 | Ti4+ | Fe2+ |
| 29 | 0.00703 | Ru4+ | Fe3+ |
| 30 | 0.006992 | Rh3+ | Fe2+ |
| 31 | 0.006648 | Bi5+ | Fe2+ |
| 32 | 0.006648 | Y3+ | Fe2+ |
| 33 | 0.006648 | Ti3+ | Fe2+ |
| 34 | 0.006648 | Ir4+ | Fe2+ |
| 35 | 0.005982 | Ce4+ | Fe3+ |
| 36 | 0.005982 | Ru5+ | Fe3+ |
| 37 | 0.005838 | Er3+ | Fe2+ |
| 38 | 0.005838 | Ni3+ | Fe2+ |
| 39 | 0.005838 | Tm3+ | Fe2+ |
| 40 | 0.005366 | V4+ | Fe2+ |
| 41 | 0.005366 | Nb5+ | Fe2+ |
| 42 | 0.005348 | Pr4+ | Fe3+ |
| 43 | 0.004846 | Dy3+ | Fe2+ |
| 44 | 0.004831 | Nb4+ | Fe3+ |
| 45 | 0.004806 | Br- | O2- |
| 46 | 0.004679 | Ge4+ | Fe3+ |
| 47 | 0.004287 | Tb3+ | Fe2+ |
| 48 | 0.004287 | Mn3+ | Fe2+ |
